# Discriminating mild from critical COVID-19 by innate and adaptive immune single-cell profiling of bronchoalveolar lavages

**DOI:** 10.1038/s41422-020-00455-9

**Published:** 2021-01-21

**Authors:** Els Wauters, Pierre Van Mol, Abhishek Dinkarnath Garg, Sander Jansen, Yannick Van Herck, Lore Vanderbeke, Ayse Bassez, Bram Boeckx, Bert Malengier-Devlies, Anna Timmerman, Thomas Van Brussel, Tina Van Buyten, Rogier Schepers, Elisabeth Heylen, Dieter Dauwe, Christophe Dooms, Jan Gunst, Greet Hermans, Philippe Meersseman, Dries Testelmans, Jonas Yserbyt, Sabine Tejpar, Walter De Wever, Patrick Matthys, CONTAGIOUS collaborators, Maria Bosisio, Michael Casaer, Frederik De Smet, Paul De Munter, Stephanie Humblet-Baron, Adrian Liston, Natalie Lorent, Kim Martinod, Paul Proost, Jeroen Raes, Karin Thevissen, Robin Vos, Birgit Weynand, Carine Wouters, Johan Neyts, Joost Wauters, Junbin Qian, Diether Lambrechts

**Affiliations:** 1 Laboratory of Respiratory Diseases and Thoracic Surgery (BREATHE), Department of Chronic Diseases and Metabolism, KU Leuven, Leuven, Belgium; 2 Department of Pneumology, University Hospitals Leuven, Leuven, Belgium; 3 Laboratory of Translational Genetics, Department of Human Genetics, KU Leuven, Leuven, Belgium; 4 VIB Center for Cancer Biology, VIB, Leuven, Belgium; 5 Laboratory for Cell Stress & Immunity (CSI), Department of Cellular and Molecular Medicine (CMM), KU Leuven, Leuven, Belgium; 6 Laboratory of Virology and Chemotherapy, Department of Microbiology, Immunology and Transplantation, Rega Institute, KU Leuven, Leuven, Belgium; 7 Laboratory of Experimental Oncology, Department of Oncology, KU Leuven, Leuven, Belgium; 8 Laboratory of Clinical Bacteriology and Mycology, Department of Microbiology, Immunology and Transplantation, KU Leuven, Leuven, Belgium; 9 Laboratory of Immunobiology, Department of Microbiology, Immunology and Transplantation, Rega Institute, KU Leuven, Leuven, Belgium; 10 Laboratory of Intensive Care Medicine, Department of Cellular and Molecular Medicine, KU Leuven, Leuven, Belgium; 11 Laboratory for Clinical Infectious and Inflammatory Disorders, Department of Microbiology, Immunology and Transplantation, KU Leuven, Leuven, Belgium; 12 Molecular Digestive Oncology, Department of Oncology, KU Leuven, Leuven, Belgium; 13 Department of Imaging & Pathology, KU Leuven, Leuven, Belgium; 14 Department of Gynecologic Oncology, Women’s Hospital, Zhejiang University School of Medicine, Hangzhou, Zhejiang 310006 China; 15 Translational Cell & Tissue Research, Department of Imaging & Pathology, KU Leuven, Leuven, Belgium; 16 Laboratory for Precision Cancer Medicine, Translational Cell and Tissue Research, Department of Imaging & Pathology, KU Leuven, Leuven, Belgium; 17 Department of Microbiology and Immunology, KU Leuven and Clinical Department of General Internal Medicine, University Hospitals Leuven, Leuven, Belgium; 18 Adaptive Immunology, Department of Microbiology, Immunology and Transplantation, KU Leuven, Leuven, Belgium; 19 Laboratory of Lymphocyte Signalling and Development, The Babraham Institute, Babraham Research Campus, Cambridge, United Kingdom; 20 Centre for Molecular and Vascular Biology, Department of Cardiovascular Sciences, KU Leuven, Leuven, Belgium; 21 Molecular Immunology (Rega Institute), Department of Microbiology, Immunology and Transplantation, KU Leuven, Leuven, Belgium; 22 Laboratory of Molecular Bacteriology (Rega Institute), Department of Microbiology, Immunology and Transplantation, KU Leuven, Leuven, Belgium; 23 Centre of Microbial and Plant Genetics, Department of Microbial and Molecular Systems (MS), KU Leuven, Leuven, Belgium; 24 Immunobiology, Department of Microbiology, Immunology and Transplantation, Rega Institute, KU Leuven, Leuven, Belgium

**Keywords:** Genome-wide analysis of gene expression, Innate immunity, Bioinformatics

## Abstract

How the innate and adaptive host immune system miscommunicate to worsen COVID-19 immunopathology has not been fully elucidated. Here, we perform single-cell deep-immune profiling of bronchoalveolar lavage (BAL) samples from 5 patients with mild and 26 with critical COVID-19 in comparison to BALs from non-COVID-19 pneumonia and normal lung. We use pseudotime inference to build T-cell and monocyte-to-macrophage trajectories and model gene expression changes along them. In mild COVID-19, CD8^+^ resident-memory (T_RM_) and CD4^+^ T-helper-17 (T_H17_) cells undergo active (presumably antigen-driven) expansion towards the end of the trajectory, and are characterized by good effector functions, while in critical COVID-19 they remain more naïve. Vice versa, CD4^+^ T-cells with T-helper-1 characteristics (T_H1_-like) and CD8^+^ T-cells expressing exhaustion markers (T_EX_-like) are enriched halfway their trajectories in mild COVID-19, where they also exhibit good effector functions, while in critical COVID-19 they show evidence of inflammation-associated stress at the end of their trajectories. Monocyte-to-macrophage trajectories show that chronic hyperinflammatory monocytes are enriched in critical COVID-19, while alveolar macrophages, otherwise characterized by anti-inflammatory and antigen-presenting characteristics, are depleted. In critical COVID-19, monocytes contribute to an ATP-purinergic signaling-inflammasome footprint that could enable COVID-19 associated fibrosis and worsen disease-severity. Finally, viral RNA-tracking reveals infected lung epithelial cells, and a significant proportion of neutrophils and macrophages that are involved in viral clearance.

## Introduction

SARS-CoV-2 has rapidly swept across the globe affecting > 33 million people, with > 1 million fatal cases.^1^ It is now well appreciated that while most COVID-19 patients (80%) remain asymptomatic or experience only mild symptoms, 20% present with overt pneumonia; about a quarter of these progressing to life-threatening Acute Respiratory Distress Syndrome (ARDS) and severe or atypical systemic inflammation.^2^ Fever, increased acute phase reactants and coagulopathy with decreased lymphocyte counts, pronounced myeloid inflammation and increased neutrophil-to-lymphocyte ratio are predominant immunological hallmarks of critical COVID-19.^3,4^

Wen et al. were the first to provide an immune atlas of circulating mononuclear cells from 10 COVID-19 patients based on single-cell RNA-sequencing (scRNA-seq). Lymphocyte counts were globally decreased, while inflammatory myeloid cells, predominantly IL1β-secreting classical monocytes, were more abundant, suggesting COVID-19 immunopathology to be a myeloid-driven process.^5^ Meanwhile, at least 8 other studies have used scRNA-seq to characterize the peripheral adaptive immune response to SARS-CoV-2,^6–13^ consistently confirming an enrichment of classical monocytes in critical COVID-19.^6–11^ Additionally, several studies reported an increase in dysfunctional neutrophils, especially in critical disease.^6–9^ With respect to interferon (IFN) signaling the situation is less clear, with several studies identifying reduced IFN signaling as a distinguishing feature of critical disease,^7–9^ compared to other studies reporting on exaggerated IFN-driven inflammation in critical vs mild COVID-19.^10,11^

However, profiling the peripheral immune landscape in COVID-19 may not be as comprehensive since immune characteristics in the periphery are different from those within the lungs, both in terms of amplitude and qualitative characteristics, as well as duration of the immune response. Thus, a better understanding of the immune interactions in COVID-19 lungs is needed. In their seminal paper, Liao et al. applied single-cell T-cell receptor-sequencing (scTCR-seq) and scRNA-seq on BAL from 3 mild and 6 critical COVID-19 patients, as well as 3 healthy controls. They observed an abundance of highly inflammatory monocytes and neutrophils and T-cell depletion in critical COVID-19. In mild COVID-19, a more potent adaptive immune response to SARS-CoV-2 was observed, as evidenced by the presence of CD8^+^ T-cells with tissue-resident features displaying clonal expansion and increased effector function.^14^ Subsequently, Bost et al. monitored viral sequencing reads at single-cell level to separate infected from bystander cells and investigated virus-induced transcriptional changes. They showed that epithelial cells are the main target of SARS-CoV-2, while viral RNA was also observed in macrophages.^15^ It was unclear however whether this represents direct viral infection of myeloid cells, or phagocytosis of viral particles (or virus-infected cells). Finally, Chua et al. performed scRNA-seq on upper and lower respiratory samples from 19 COVID-19 patients, supporting the notion that a balanced cytotoxic T-cell signature (expressing perforins, granzymes, interferons, *etc*.) defines an effective immune response against SARS-CoV-2.^16^ In summary, while scRNA-seq on COVID-19 pneumonia BAL already contributed to important hypothesis-generating datasets, in-depth characterization of the immunological mechanisms underlying mild vs critical COVID-19 remains largely unexplored, mostly due to small sample sizes analyzed. Additionally, analyses so far did not include BAL samples of relevant control groups such as non-COVID-19 pneumonia cases.

Here, we provide a comprehensive deep-immune atlas of COVID-19 pneumonia, analyzing BAL from 31 COVID-19 patients with mild or critical disease, while inclusion of 13 patients with non-COVID-19 pneumonia allowed us to reliably distinguish non-specific lung-localized inflammatory signaling from COVID-19-specific lung-associated immune changes.

## Results

### ScRNA-seq and cell typing of BAL samples

We performed scRNA-seq on BAL from 22 hospitalized patients with a positive qRT-PCR for SARS-CoV-2 on a nasopharyngeal swab or a lower respiratory tract sample. We also collected BAL from 13 patients with clinical suspicion of COVID-19 pneumonia, yet negative PCR on lower respiratory tract sampling for SARS-CoV-2. These samples are referred to as non-COVID-19 and comprise both bacterial and *Pneumocytis Jirovecii* pneumonia cases. We further stratified patients by disease severity at the time of sampling, by discerning two groups; a ‘mild’ and a ‘critical’ disease group, the latter requiring high-flow oxygen therapy, mechanical ventilation or extracorporeal membrane oxygenation. Demographic and clinical data of the prospectively recruited patient cohort, including co-morbidities and computed tomography to quantify lung injury are summarized in Supplementary information, Table S1.

**Fig. 1:**
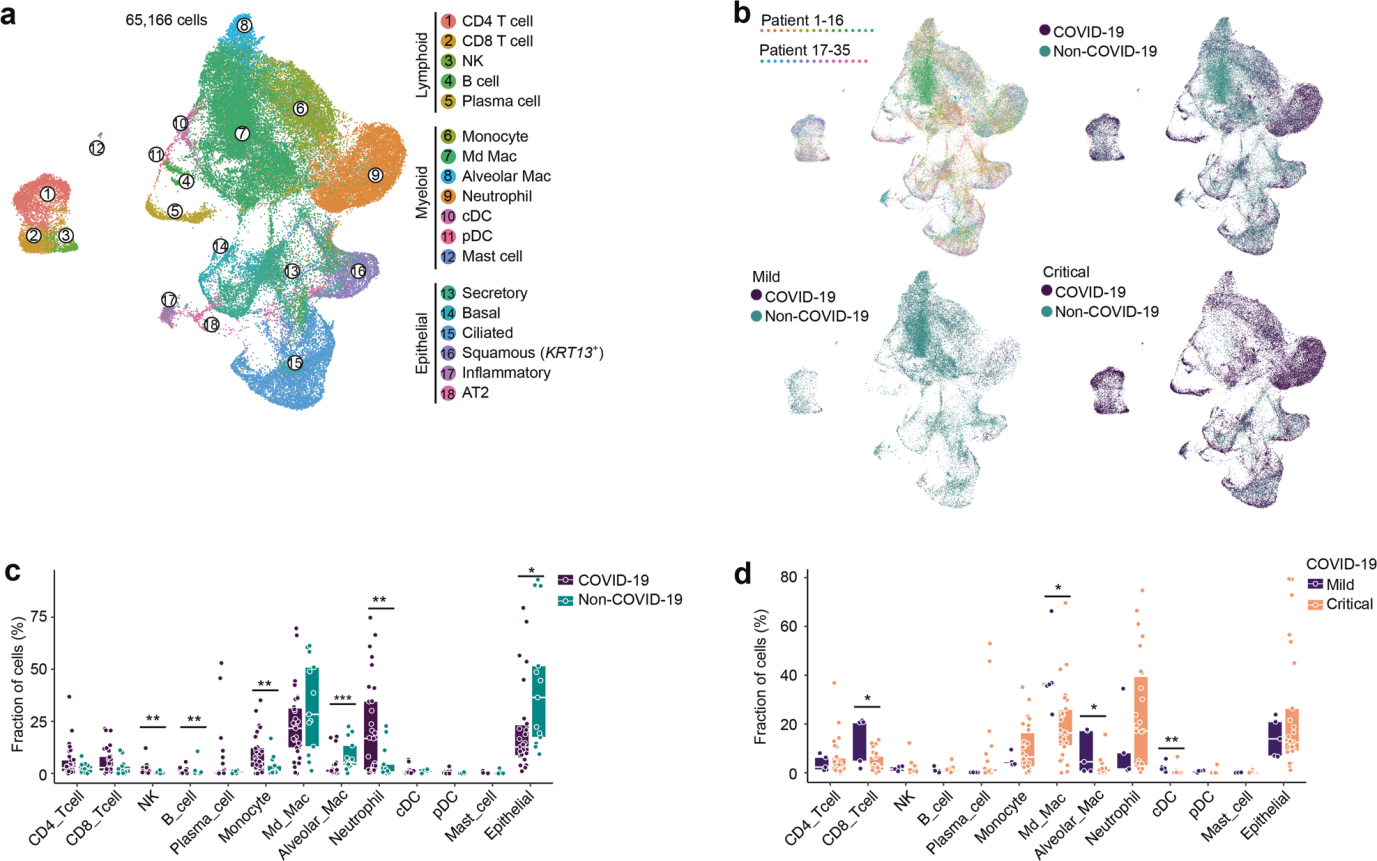
Annotation of cell types by scRNA-seq in COVID-19 and non-COVID-19 BAL. **a** UMAP representation of 65,166 cells (obtained from BAL from *n* = 13 non-COVID-19, *n* = 2 mild and *n* = 22 critical COVID-19 patients) by scRNA-seq color-coded for the indicated cell type. pDC, plasmacytoid dendritic cell; cDC, conventional dendritic cell; NK, natural-killer cell; Md_Mac, monocyte-derived macrophage; Alveolar_Mac, alveolar macrophage; AT2, alveolar type II epithelial cell. **b** UMAP panels stratified per individual patient, COVID-19 vs non-COVID-19 and mild vs critical COVID-19. **c** Relative contribution of each cell type (in %) in COVID-19 vs non-COVID-19. **d** Relative contribution of each cell type (in %) in mild vs critical COVID-19. *P* values were assessed by Mann–Whitney test. *P < 0.05, **P < 0.01, ***P < 0.001. After correction for disease severity, gender, age and underlying disorders (hypertension and type II diabetes), only neutrophils and epithelial cells differed significantly (*P* = 0.031 and *P* = 0.014, respectively).

BAL samples were immediately processed for scRNA-seq. After quality filtering (Materials and Methods), we obtained ~186 million of unique transcripts from 65,166 cells with > 150 genes detected. Of these, ~51% of cells were from COVID-19. Subsequent analysis involving dimensionality reduction and clustering identified several clusters (Fig. 1a), which through marker genes (Supplementary information, Fig. S1a–c) could be assigned to lymphoid cells (CD4^+^ and CD8^+^ T-cells, natural killer cells (NK), B-cells and plasma cells), myeloid cells (monocytes/macrophages, neutrophils, mast cells, plasmacytoid dendritic cells/pDCs and conventional dendritic cells/cDCs) and epithelial cells (including secretory, basal, ciliated, squamous, inflammatory and AT2 lung epithelial cells). We describe each cell type in more detail, highlighting the number of cells, read counts and transcripts detected in Supplementary information, Table S2. There was no cluster bias between disease status (COVID-19 vs non-COVID-19), disease severity (mild vs critical) or individual patients (Fig. 1b; Supplementary information, Fig. S1c).

To increase our resolution, we processed scRNA-seq data on COVID-19 BAL by Liao et al., consisting of 3 patients with ‘mild’ and 6 patients with ‘critical’ COVID-19 (*n* = 51,631 cells) (Supplementary information, Fig. S1d).^14^ We also retrieved 7 normal lung samples (*n* = 64,876 cells) profiled by Lambrechts et al. and 8 normal lung samples (*n* = 27,266 cells) by Reyfman et al. to further enhance our resolution (specifically for T-cells and DCs).^17,18^ Datasets were integrated by clustering cells from each dataset separately and assigning cell type identities to each cell. We then pooled cells from each dataset based on cell type identities and performed canonical correlation analysis (CCA), as described previously,^19^ followed by graph-based clustering to generate a UMAP per cell type, displaying its phenotypic heterogeneity. Using a separate cluster strategy (see Materials and Methods) we found that > 93% of cells clustered similarly, indicating robostness of the clustering approach (Supplementary information, Fig. S1e–g). Notably, since neutrophils are important during COVID-19, but are low in RNA content and therefore often missed in scRNA-seq data, we lowered our filtering thresholds removing cells with < 151 genes (instead of < 201 genes) expressed. This led to a relative increase in the number of neutrophils detected (Supplementary information, Fig. S1h).

After integration, data were derived from 5 mild and 26 critical COVID-19 patients, and compared to 10 mild and 3 critical non-COVID-19 patients. Quantitatively, monocyte/macrophages and neutrophils were the most abundant cell types, amounting up to 65.7% (*n* = 55,825) of COVID-19 cells (Fig. 1c). When evaluating the relative enrichment or depletion of these cell types, we found that monocytes and neutrophils were more frequent in COVID-19 vs non-COVID-19 patients, especially in critical patients (Fig. 1c; Supplementary information, Fig. S1i). On the other hand, macrophages and epithelial cells were less abundant in COVID-19, especially in critical patients. CD8^+^ T-cells and NK-cells were slightly enriched and this mostly in mild disease. When comparing mild vs critical COVID-19, an increase in CD8^+^ T-cells, macrophages and cDCs was noticed in the former (Fig. 1d). These changes were largely maintained when comparing mild vs critical COVID-19 separately for both cohorts (Liao et al.^14^ vs this study, Supplementary information, Table S3).

Below, we describe the heterogeneity underlying each cell type in more detail. A list of marker genes used to identify each cellular phenotype is highlighted in Supplementary information, Table S4.

### Phenotypic heterogeneity of CD8^+^ T-cells in COVID-19 BAL

**Fig. 2:**
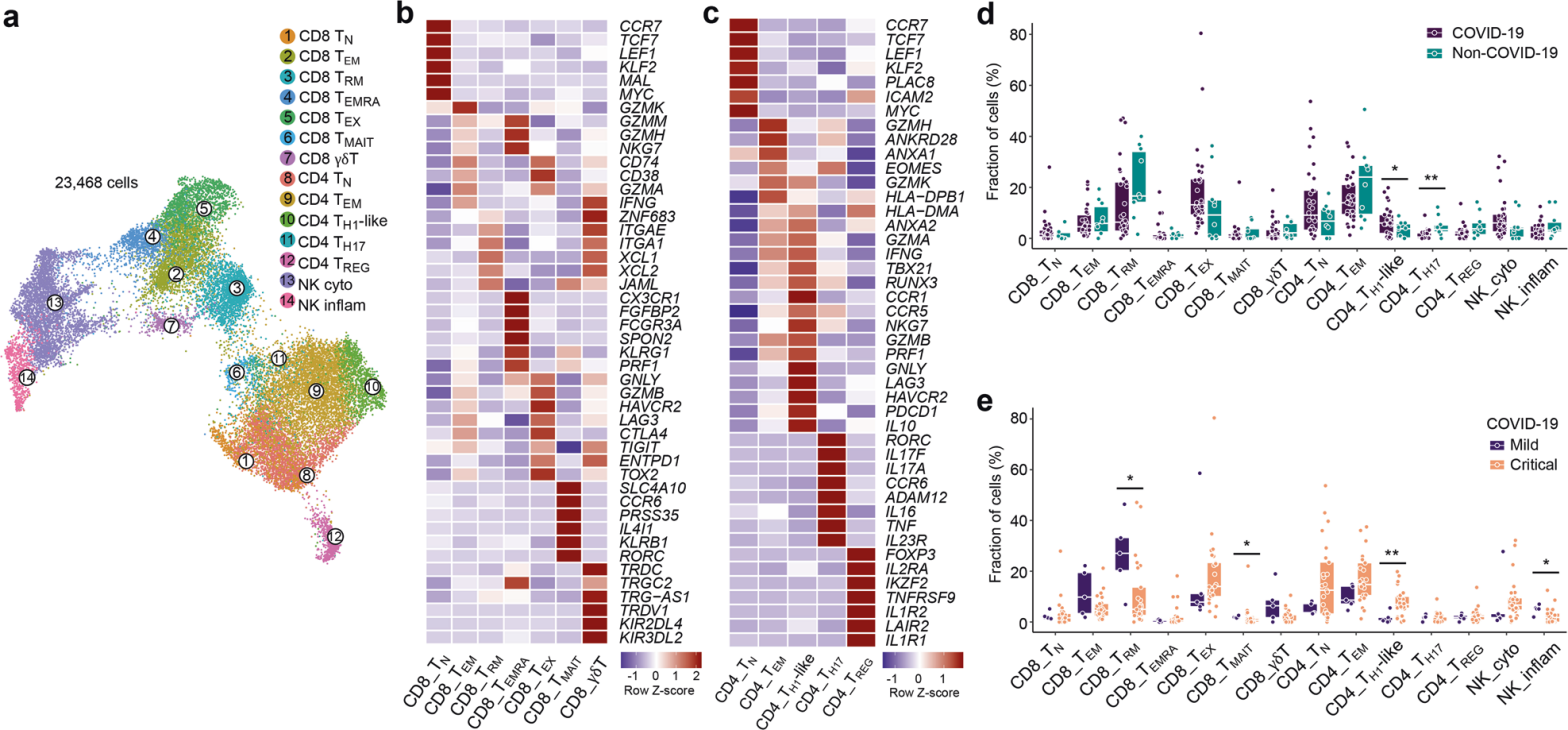
14 T-cell phenotypes in mild and critical COVID-19 BAL. **a** Subclustering of 23,468 T-/NK-cells into 14 T-/NK-cell phenotypes, as indicated by the color-coded legend. NK_cyto, cytotoxic NK cell; NK_inflam, inflammatory NK cell. **b, c** Heatmap showing marker genes for CD8^+^ (**b**) and CD4^+^ (**c**) T-cell phenotypes. **d** Relative contribution of each T-/NK-cell phenotype (in %) in COVID-19 vs non-COVID-19. **e** Relative contribution of each T-/NK-cell phenotype (in %) in mild vs critical COVID-19. *P* values were assessed by Mann–Whitney test. *P < 0.05, **P < 0.01, ***P < 0.001.

Altogether, we retrieved 23,468 T- and NK-cells, which were subclustered into 14 phenotypes (Fig. 2a–c; Supplementary information, Fig. S2a–c). To avoid that proliferative T-cells would cluster separately, we regressed for cell cycle genes in this analysis (see Materials and Methods). Overall, we identified 7 CD8^+^ T-cell clusters, 5 CD4^+^ T-cell clusters and 2 NK-cell clusters. While naïve CD8^+^ T-cells (T_N_) expressed naïve T-cell markers (*CCR7*, *LEF1* and *TCF7*), effector-memory (T_EM_; *GZMK*, *GZMH*, *GZMM*) and exhaustion-like T-cells (T_EX_) were characterized by increased expression of effector markers (*GZMA*, *GNLY*, *GZMB*, *IFNG*). Herein, expression of (inflammation-driven) exhaustion-defining immune-checkpoints (*HAVCR2*, *CTLA4*, *LAG3*) distinguished T_EX_-cells. Additionally, we identified CD8^+^ resident-memory T-cells (T_RM_) based on *ZNF683* and *ITGAE*, as well as CD8^+^ recently-activated effector-memory T-cells (T_EMRA_; *CX3CR1*, *FGFBP2*, *FCGR3A*). Finally, we also identified mucosal-associated invariant (T_MAIT_; *SLC4A10*, *PRSS35*, *CCR6*) and gamma-delta (T_γδ_; *TRDC*, *TRGC2* and *TRG-AS1*) T-cells.

Next, we assessed prevalence of each T-cell phenotype in COVID-19 vs non-COVID-19 disease, but failed to observe differences in the CD8^+^ phenotypes (Fig. 2d; Supplementary information, Fig. S2d). When comparing mild to critical COVID-19 (Fig. 2e), we found T_MAIT_-cells to be slightly increased in the former. Interestingly, T_MAIT_-cells can actively co-opt for specific innate immune characteristics (e.g., proficient pattern-recognition receptor-based signaling, and/or broad non-MHC antigenic surveillance), thereby allowing them to rapidly respond to pathogenic agents possessing pathogen-associated molecular patterns (PAMPs).^20^

**Fig. 3:**
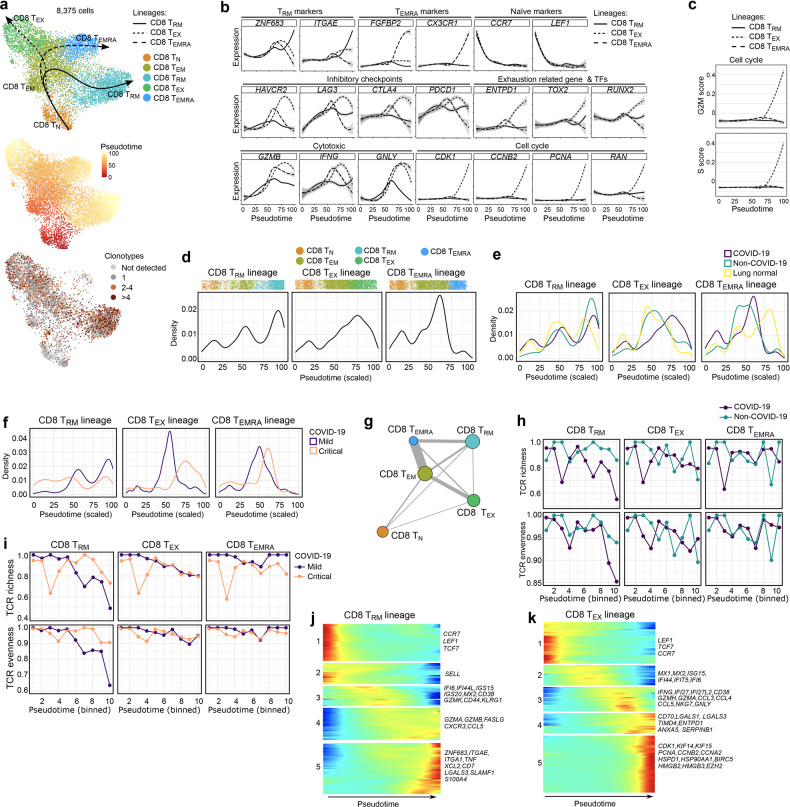
CD8^+^ T-cell phenotypes in mild and critical COVID-19 BAL. **a** Pseudotime trajectories for CD8^+^ T-cells based on Slingshot, showing 3 lineages (T_RM_-lineage, T_EX_-lineage and T_EMRA_-lineage), color-coded for the CD8^+^ T-cell phenotypes (upper panel), the pseudotime (middle panel) and the number of clonotypes (lower panel). Since no scTCR-seq data were available from healthy lung tissue, the number of T_EMRA_-cells is very low in the clonotype analysis. **b** Profiling of marker and functional genes along these trajectories to confirm their functional annotation. **c** Dynamics of T-cell proliferation along the CD8^+^ T-cell lineages are depicted by plotting cell cycle G2M and S scores. **d** Density plots reflecting the number of T-cells along the 3 CD8^+^ T-cell lineages. **e** Density plots reflecting the number of T-cells along the 3 CD8^+^ T-cell lineages stratified for non-COVID-19, COVID-19 and normal lung. **f** Density plots reflecting the number of T-cells along the 3 CD8^+^ T-cell lineages stratified for mild vs critical COVID-19. **g** Analysis of clonotype sharing (thickness indicates proportion of sharing, circle size indicates clonotype counts) between the CD8^+^ T-cells. **h, i** TCR richness and TCR evenness along the 3 T-cell lineages for non-COVID-19 vs COVID-19 (**h**), and mild vs critical COVID-19 (**i**). **j, k** Gene expression dynamics along the CD8^+^ T_RM_- (**j**) and T_EX_-lineage (**k**). Genes cluster into 5 gene sets, each of them characterized by specific expression profiles, as depicted by a selection of marker gene characteristic for each set. Differences in trajectories were assessed by Mann–Whitney test. For CD8^+^ T_RM_: COVID-19 vs non-COVID-19 (*P* = 1.0E-6), mild vs critical COVID-19 (*P* = 5.9E-102). For CD8^+^ T_EX_: COVID-19 vs non-COVID-19 and normal lung (*P* = 2.3E-67), mild vs critical (*P* = 1.1E-39). For CD8^+^ T_EMRA_: normal lung vs COVID-19 and non-COVID-19 (*P* = 3.8E-39).

The largest increase in mild vs critical COVID-19, however, was seen for CD8^+^ T_RM_-cells. To understand this difference, we used Slingshot to infer pseudotime trajectories (excluding T_MAIT_- and T_γδ_-cells). We observed 3 distinct trajectories (Fig. 3a): CD8^+^ T_N_-cells connected with T_EM_-cells, which subsequently branched into 3 different (well-connected) lineages i.e., T_RM_-cells, T_EX_-cells and T_EMRA_-cells, with nearly all CD8^+^ T_N_-cells (99.6%) shared by all 3 trajectories (Supplementary information, Fig. S2e). Profiling of marker genes, inhibitory checkpoints, cytotoxic markers and proliferation along these trajectories confirmed their functional annotation (Fig. 3b, c). Notably, besides increasing inhibitory checkpoint and cytotoxic marker expression, CD8^+^ T_EX_-cells were characterized by proliferation (Supplementary information, Fig. S2f) with G2M and S gene scores progressively increasing along the trajectory (Fig. 3b, c). Next, density plots reflecting the relative number of T-cells in each phenotypic state were created along these trajectories (Fig. 3d), and stratified for normal tissue, non-COVID-19, and mild or critical COVID-19. Non-COVID-19 T-cells were enriched towards the end of the T_RM_-lineage, while in COVID-19 this was the case for the T_EX_-lineage (Fig. 3e). In contrast, at the end of the T_EMRA_-lineage cells were enriched for normal lung. When comparing mild to critical COVID-19, cells from the T_RM_-lineage were enriched at the end of the lineage in mild COVID-19, while along the T_EX_-lineage such enrichment was most prominent for critical COVID-19 (Fig. 3f). There were no obvious differences in the T_EMRA_-lineage.

We also processed T-cells by scTCR-seq, obtaining 3966 T-cells with a TCR sequence that were also annotated by scRNA-seq (excluding NK-, T_MAIT_- and T_γδ_-cells). Based on TCR sharing, we could reinforce the 3 trajectories identified by Slingshot (Fig. 3g). Overall, CD8^+^ T_RM_-cells contained the highest number of T-cell clonotypes. Plotting TCR richness and evenness along the trajectories, revealed that both parameters were reduced along the T_RM_-lineage, specifically in COVID-19 (Fig. 3h), likely indicating antigen-driven clonal expansion. Notably, this expansion was more prominent in mild COVID-19 (Fig. 3i). In contrast, in the T_EMRA_- and T_EX_-lineage, richness did not decrease along the pseudotime.

Overall, this suggests that mild COVID-19 is characterized by T_RM_-cells undergoing active (presumably antigen-driven) TCR expansion and selection at the end of the trajectory, while T_EX_-cells are enriched halfway their trajectory. In critical COVID-19, both T_RM_- and T_EX_-cells fail to undergo expansion, despite the latter being located mainly at the end of the lineage.

### Gene expression modelling along the CD8^+^ T_RM_- and T_EX_-lineage

We then modelled gene expression along the T_RM_- and T_EX_-lineage, and identified 5 gene sets with specific expression patterns in each trajectory. In the T_RM_-lineage, set 1 and 2 consisted of naïve T-cell markers (set 1: *CCR7*, *LEF1, TCF7*; set 2: *SELL*), whose expression decreased along the trajectory (Fig. 3j; Supplementary information, Table S5). A third set was enriched for IFN-induced (anti-viral) genes (*IFI6*, *IFI44L*, *ISG15*, *ISG20*, *MX2*), activation-associated genes (*CD38*) and genes mediating effector-memory functions (*GZMK, CD44, KLRG1*). These genes exhibited high expression halfway the trajectory. Genes from the last 2 sets were expressed at the end of the trajectory and consisted of cytotoxic or increased effector function genes (set 4: *GZMA, GZMB, FASLG, CXCR3, CCL5*), pro-inflammatory and auto-regulatory genes (set 5: *ITGA1*, *TNF*, *XCL2, CD7* and *LGALS3*, *SLAMF1*, *S100A4*) and genes marking resident-memory formation (*ZNF683, ITGAE*).^21,22^ In mild COVID-19, T_RM_-cells mainly expressed set 3–5 genes, indicating increased (but balanced) effector function (Supplementary information, Fig. S2g, h), while in critical COVID-19 T_RM_-cells expressed set 1–2 genes, indicating a more naïve state. Indeed, mild COVID-19 patients had a significantly higher ‘resident-memory’ effector score (*P* = 0.016), but lower naïve T-cell score (*P* = 0.049) compared to critical COVID-19 (Supplementary information, Fig. S2i).

In the T_EX_-lineage, the first gene set contained naïve T-cell markers (*LEF1*, *CCR7*, *TCF7*), a second IFN-induced (anti-viral) genes (*MX1*, *MX2, ISG15*, *IFI44*, *IFIT5*, *IFI6*), while a third besides *IFNG* and IFN-induced genes (*IFI27*, *IFI27L2*) was comprised of T-cell activation-related genes (*CD38*, *GZMH*, *GZMA*), chemokines (*CCL3*, *CCL4* and *CCL5*), cytotoxicity- (*NKG7*, *GNLY*, *GZMB*) and (inflammatory) exhaustion-related genes (*HAVCR2*) (Fig. 3k; Supplementary information, Table S6). Set 4 was characterized by expression of pro-inflammatory (*CD70*, *COTL*, *HMGB1*) and anti-inflammatory genes (*ENTPD1*, *ANXA5*, *SERPINB1*), suggesting that these cells exhibit a chronic dysregulated hyperinflammatory phenotype. We also noticed expression of the TIM auto-regulatory protein family (*TIMD4*) and viral infection-induced auto-regulatory genes (*LGALS1*, *LGALS3*).^23,24^ In set 5, cell-cycle genes (*CDK1*, *KIFs*, *PCNA*, *CCNA/B2*), stress-associated genes (*HSPD1*, *HSP90AA1*, *BIRC5*) and chromatin remodeling-related genes (*HMGB2*, *HMGB3*, *EZH2*) were increased, suggesting that T-cells were largely adjusting to inflammation-driven stress (rather than mounting any discernible effector or auto-regulatory responses). Notably, mild COVID-19 T_EX_-cells exhibited increased expression of set 2-associated genes, while critical COVID-19 T_EX_-cells had increased expression of set 4–5 genes (Supplementary information, Fig. S2j).

Overall, gene expression profiling along the trajectories confirmed that mild COVID-19 exhibits CD8^+^ T_RM_- and T_EX_-cells with good effector function, while in critical COVID-19 this effector function is drastically reduced possibly due to (persistent) inflammation-associated stress.

### Phenotypic heterogeneity of CD4^+^ T-cells in COVID-19 BAL

We identified naïve CD4^+^ T-cells (T_N_; *CCR7*, *TCF7*, *LEF1*), effector-memory T-cells (T_EM_; *GZMH*, *GZMK*, *ANXA1*), CD4^+^ T-helper-1-like (T_H1_-like) cells, expressing T_H1_-like transcription factors (*TBX21*, *RUNX3*), immune-checkpoints (*HAVCR2*, *LAG3*, *PDCD1* and *CTLA4*) and cytotoxic genes (*NKG7*, *GZMB*, *GNLY*, *PRF1*), as well as CD4^+^ T-helper-17 (T_H17_; *RORC*, *IL17A/F*, *CCR6*, *IL23R*) and CD4^+^ regulatory T-cells (T_REG_; *FOXP3*, *IL2RA*, *IKZF2*) (Fig. 2c; Supplementary information, Fig. S2b). Compared to non-COVID-19, we observed slightly less CD4^+^ T_H17_-cells, but more T_H1_-like-cells in COVID-19. Comparing mild vs critical COVID-19, we found T_H1_-like-cells to be significantly increased in the latter (Fig. 2d, e).

**Fig. 4:**
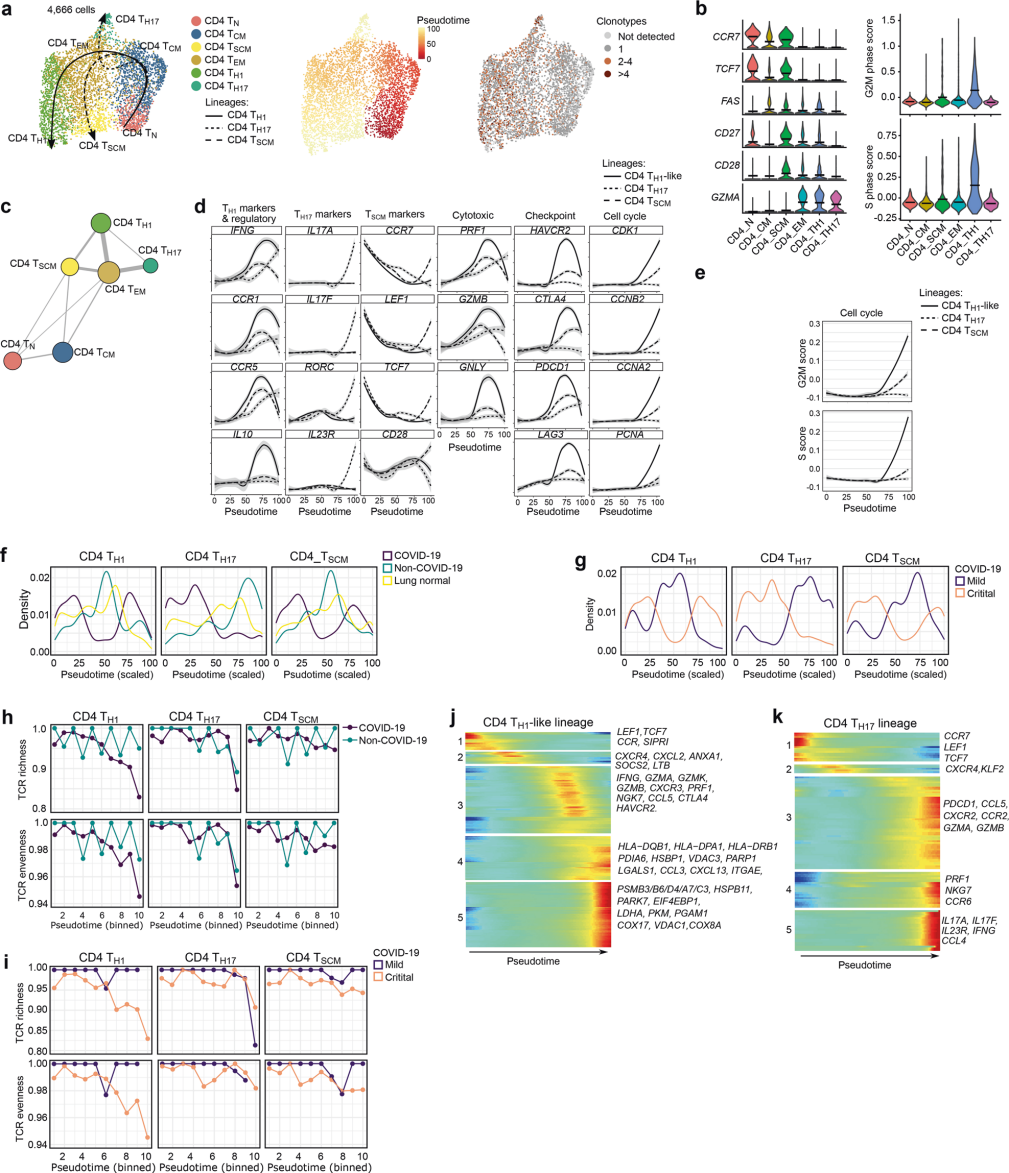
CD4^+^ T-cell developmental trajectories in mild and critical COVID-19 BAL. **a** UMAP with pseudotime trajectories based on Slingshot, showing 3 lineages (T_H1_-lineage, T_H17_-lineage and T_SCM_-lineage), color-coded for the CD4^+^ T-cell phenotypes (left), the pseudotime (middle) and the number of clonotypes (right). T_SCM_-cells represent a subset of minimally differentiated T-cells characterized by phenotypic and functional properties that bridge naïve and conventional memory T-cells.^81^ T_SCM_-cells include cells that show high expression of naïve (or precursor) markers (*CCR7*, *TCF7*), but not of activation markers (*GZMA*), high expression of memory markers (*CD27*, *CD28* and *CD95*), are more proliferative compared to T_N_- and T_CM_-cells (increased G2M and S scores), increased TCR clonotype expansion compared to T_N_- and T_CM_-cells. **b** Naïve and memory-related marker gene expression (left), and cell cycle scoring (right) reveal additional CD4^+^ T-cell subclusters. T_SCM_-cells are characterized by naïve marker genes (*CCR7*, *TCF7*), memory markers (*CD27*), cell proliferation but no *GZMA* expression. **c** Analysis of clonotype sharing (thickness indicates proportion of sharing, circle size indicates clonotype counts) between the CD4^+^ T-cell subclusters. **d** Profiling of marker and functional genes along these trajectories to confirm their functional annotation. **e** Dynamics of T-cell proliferation along the CD4^+^ T-cell lineages are depicted by plotting cell cycle G2M and S scores. **f** Density plots reflecting the number of T-cells along the 3 CD4^+^ T-cell lineages stratified for non-COVID-19, COVID-19 and normal lung. **g** Density plots reflecting the number of T-cells along the 3 CD4^+^ T-cell lineages stratified for mild vs critical COVID-19. **h, i** TCR richness and TCR evenness along the 3 CD4^+^ T-cell lineages comparing non-COVID-19 vs COVID-19 (**h)** and mild vs critical COVID-19 (**i**). **j, k** Gene expression dynamics along the CD4^+^ T_H1_- (**j**) and T_H17_-lineage (**k**). Genes cluster into 5 gene sets, each of them characterized by specific expression profiles, as depicted by a selection of marker genes characteristic for each set. Differences in trajectories were assessed by Mann–Whitney test. For CD4^+^ T_H1_ and CD4^+^ T_SCM_: COVID-19 vs non-COVID-19 and lung normal (*P* = 1.4E-6 and 5.9E-37), For CD4^+^ T_Th17_: COVID-19 vs non-COVID-19 (*P* = 9.7E-12), mild vs critical COVID-19 (*P* = 1.3E-121).

CD4^+^ T_REG_-cells could be further subclustered into two phenotypes (*TNFRSF9*^high^ and *TNFRSF9*^low^; Supplementary information, Fig. S2k, l), but due to their complex and distinct developmental process,^25^ we excluded them from subsequent trajectory analyses. Slingshot revealed additional phenotypic heterogeneity, also identifying central-memory CD4^+^ T_CM_-cells and stem cell-like memory CD4^+^ T_SCM_-cells (Fig. 4a, b). Overall, there were 3 trajectories, which were independently confirmed based on shared TCR clonotypes (Fig. 4c). Briefly, T_N_-cells connected closely with T_CM_-cells followed by T_EM_-cells, which branched-off into 3 different trajectories to form T_H1_-like, T_H17_- and T_SCM_-cells (Supplementary information, Fig. S2m). Profiling of marker genes, inhibitory checkpoints, cytotoxic and transcription factors along these trajectories confirmed their functional annotation (Fig. 4d). As reported previously,^26^ CD4^+^ T_H1_-like-cells were characterized by increased proliferation, as well as cytotoxicity and inhibitory checkpoint expression, along their trajectory (Fig. 4d, e; Supplementary information, Fig. S2f). Density plots along these trajectories revealed that COVID-19 was enriched for T-cells early and late in the T_H1_-like- and T_SCM_-trajectory, while vice versa in non-COVID-19 and normal lung they were enriched halfway these trajectories (Fig. 4f). In the T_H17_-trajectory, COVID-19 BAL was strongly enriched for T-cells in the first half of the trajectory. Overall, CD4^+^ T-cells from mild COVID-19 behaved similarly as normal lung or non-COVID-19 BAL (Fig. 4f-g). Both TCR richness and evenness were reduced along the T_H1_-like lineage from COVID-19, but not from non-COVID-19 (Fig. 4h). Notably, this reduction was most prominent in critical COVID-19 (Fig. 4i). In contrast, T_H17_-cells and T_SCM_-cells were characterized by a modest decrease in TCR richness only at the very end of their trajectory, suggesting that mainly T_H1_-like-cells are selected for specific SARS-CoV-2 PAMPs/antigens.

Overall, mild COVID-19 is characterized by T_H1_-like-cells that become entangled halfway in their trajectory, while in critical COVID-19 T_H17_-cells are stuck in the first half of their trajectory.

### Gene expression modelling along the CD4^+^ TH1- and TH17-lineage

Differential gene expression analysis along these CD4^+^ T-cell trajectories identified several gene sets. In the T_H1_-like trajectory, the first gene set consisted of naïve (*LEF1, TCF7*) and undifferentiated (*CCR7*, *S1PR1*) T-cell markers (Fig. 4j; Supplementary information, Table S7). A second set was enriched for both pro- and anti-inflammatory markers (*CXCR4*, *CXCL2*, *ANXA1*, *SOCS2*, *LTB*), while a third set was characterized (halfway the trajectory) by an effector-like T_H1_-program based on expression of *IFNG*, granzymes (*GZMA*, *GZMK*, *GZMB*), *CXCR3*, *PRF1*, *NGK7*, *CCL5*, as well as *CTLA4* and *HAVCR2*. Finally, a fourth gene set was characterized by high HLA expression, auto-regulatory markers (*LGALS1*, *CCL3*), partial activation markers (*CXCL13*) and stress-response markers (*PDIA6*, *HSBP1*, *VDAC3*, *PARP1*) at the end of the trajectory, suggesting a complex mixture of a pro- and anti-inflammatory phenotype coupled with early-stress modulation. In a final fifth set, we noticed mitochondrial stress (*LDHA*, *PKM*, *COX17, VDAC1*, *COX8A*), an IL2 withdrawal-associated stressed phenotype (*MT1E*, *MT1X*), proteotoxic stress (*PSMB3/B6/D4/A7/C3*, *HSPB11*, *PARK7*, *EIF4EBP1*) and glycolysis (*PGAM1*) suggesting ‘exhaustion’ at the end of the T_H1_-like-trajectory.^27,28^ Overall, in mild COVID-19, the T_H1_-like-lineage was characterized by increased expression of set 2 genes, indicating increased T_H1_-effector function. In critical COVID-19, expression of sets 4–5 genes pre-dominated, suggesting severe dysregulation (Supplementary information, Fig. S2n).

In the T_H17_-lineage, we also identified 5 gene sets (Fig. 4k; Supplementary information, Table S8): the first 2 sets with high expression early in the trajectory did not express markers indicative of T_H17_ function. Three other gene sets with high expression at the end of the trajectory were characterized by T-cell effector function (set 3: *PDCD1*, *CCL5*, *CXCR2*, *CCR2* and *GZMA/B*), expression of cytotoxic-activity genes (set 4: *NKG7* and *PRF1*) and T_H17_-cell associated interleukins (set 5: *IL17A*, *IL17F*, *IL23R*, as well as *IFNG* and *CCL4*). Notably, in mild COVID-19, cells were characterized by increased expression of set 3–5 genes, while critical patients exhibited expression of set 1–2 genes (Supplementary information, Fig. S2o).

Overall, this indicates that mild COVID-19 is characterized by improved T_H1_- and T_H17_-effector functions. The combined effects of T_H1_-like- and T_H17_-cells are indeed known to regulate immune responses against viral infection-associated inflammation by enhancing, balancing and regulating each other’s activities and persistence.^29,30^

### Trajectory of monocyte-to-macrophage differentiation in COVID-19 BAL

**Fig. 5:**
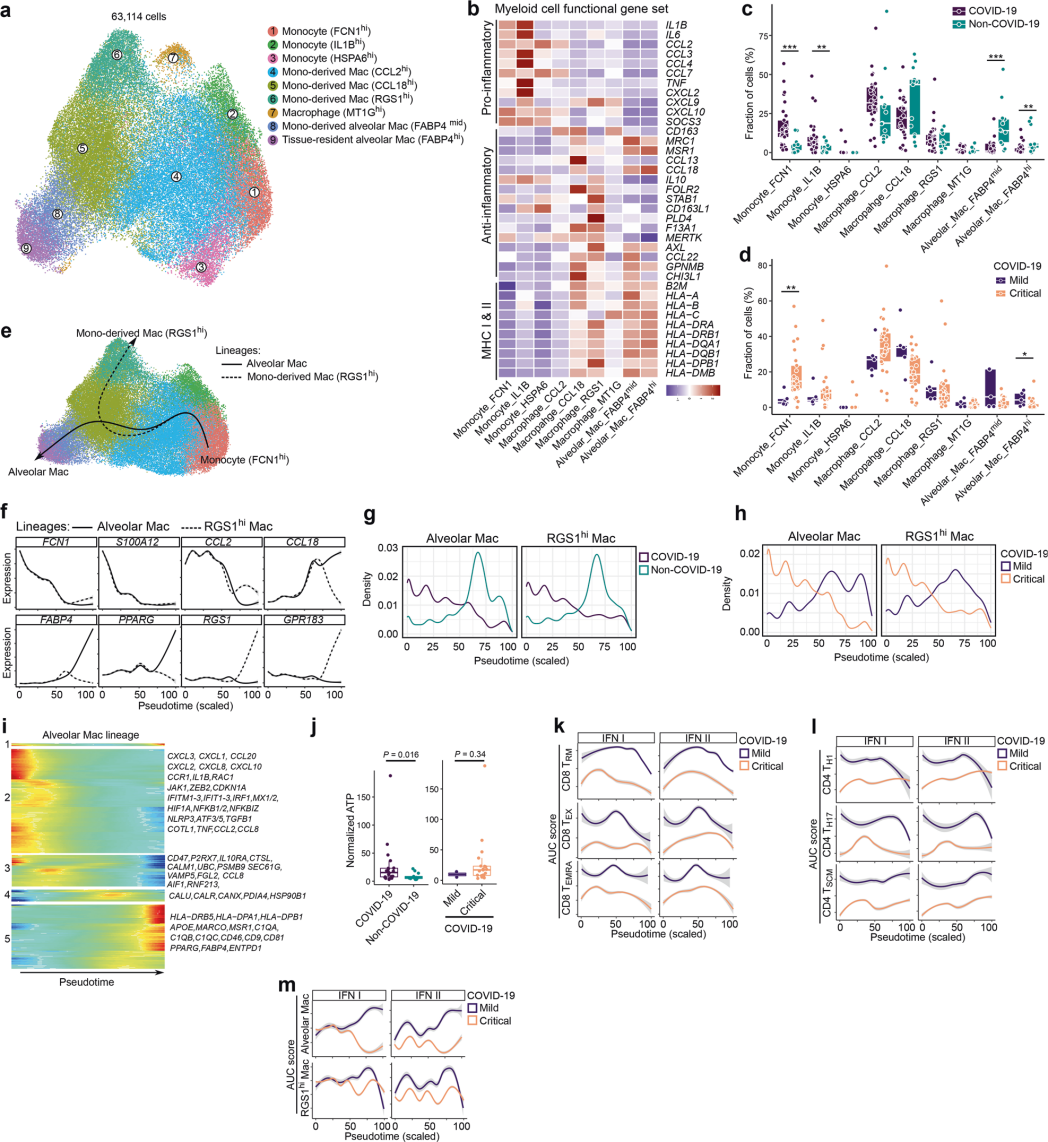
Monocyte-to-macrophage differentiation in COVID-19 BAL. **a** Subclustering of myeloid cells into 9 phenotypes, as indicated by the color-coded legend. **b** Heatmap showing myeloid cell phenotypes with corresponding functional gene sets. **c** Relative contribution of each cell type (in %) to COVID-19 vs non-COVID-19 BAL. **d** Relative contribution of each cell type (in %) to mild vs critical COVID-19 BAL. **e** Pseudotime trajectories for myeloid cells based on Slingshot, showing the common branch of FCN1^hi^ monocytes differentiating into either RGS1^hi^ monocyte-derived macrophages (RGS1^hi^-lineage) or FABP4^hi^ tissue-resident alveolar macrophages (alveolar lineage). **f** Profiling of marker genes along these trajectories to confirm their functional annotation: *FCN1, S100A12, CCL2, CCL18* for the common branch, *FABP4* and *PPARG* for the alveolar lineage, *RGS1* and *GPR183* for the RGS1-lineage. **g** Density plots reflecting the number of myeloid cells along the 2 lineages stratified for non-COVID-19 vs COVID-19. **h** Density plots reflecting the number of myeloid cells along the 2 lineages stratified for mild vs critical COVID-19. **i** Gene expression dynamics along the alveolar lineage. Genes cluster into 5 gene sets, each of them characterized by specific expression profiles, as depicted by a selection of genes characteristic for each cluster. **j** Normalized ATP level measured from BAL supernatans comparing COVID-19 vs non-COVID-19 (left) and mild vs critical COVID-19 patients (right). **k–m** Profiling of IFN type I and II signaling along the 3 CD8^+^ (**k**) and CD4^+^ (**l**) T-cell lineages, and along the monocyte-macrophage lineage (**m**), comparing mild vs critical COVID-19. All *P* values were assessed by a Mann–Whitney test. *P < 0.05, **P < 0.01, ***P < 0.001. *P* values comparing COVID-19 vs non-COVID-19, and mild vs critical COVID-19 for density plots were all < 10E-50.

In the 63,114 myeloid cells derived from BAL, we identified 9 phenotypes (Fig. 5a). Monocytes clustered separately from macrophages based on the absence of macrophage markers (*CD68*, *MSR1*, *MRC1*) and presence of monocyte markers (*IL1RN*, *FCN1*, *LILRA5*). Monocytes could be further divided into FCN1^high^, IL1B^high^ and HSPA6^high^ monocytes (Fig. 5b; Supplementary information, Fig. S3a), respectively, characterized by expression of classical monocyte markers (*IL1RN*, *S100A8/9*), pro-inflammatory cytokines (*IL1B*, *IL6*, *CCL3*, *CCL4*) and heat-shock proteins (*HSPA6*, *HSPA1A/B*). Based on *CSF1R, CSF3R* and *SPP1*, 3 monocyte-derived macrophages could further be delineated, including CCL2^high^, CCL18^high^ and RGS1^high^ macrophages (Supplementary information, Fig. S3b). CCL2^high^ clusters were characterized by the pro-migratory cytokine *CCL2*, but also by several pro- (*CCL7*, *CXCL10*) and anti-inflammatory (*CCL13*, *CCL22*) genes, suggesting existence of an intermediate population of cells. In contrast, CCL18^high^ and RGS1^high^ cells expressed mainly anti-inflammatory genes (*CCL13*, *CCL18*, *PLD4, FOLR2*), as well as genes involved in receptor-mediated phagocytosis (*MERTK*, *AXL*). Finally, we identified MT1G^high^ macrophages (expressing numerous metallothioneins suggestive of oxidative stress or immune cell’s growth factor-withdrawal), a monocyte-derived (FABP4^medium^) and tissue-resident (FABP4^high^) alveolar macrophage cluster. The latter two populations were characterized by high expression of resident markers (*FABP4*, *PPARG*), anti-inflammatory (*CCL18*, *CCL22*) and antigen-presentation relevant MHC-I/II genes.

We observed a significant increase in FCN1^high^ and IL1B^high^ monocytes in COVID-19 vs non-COVID-19, especially in critical disease (Fig. 5c; Supplementary information, Fig. S3c). Vice versa, FABP4^medium^ and FABP4^high^ alveolar macrophages were reduced in COVID-19, both in the mild and critical disease comparison, albeit non-significantly. FCN1^high^ monocytes were significantly reduced in mild vs critical COVID-19, while alveolar macrophages were increased (Fig. 5d). After excluding stress-induced phenotypes (HSPA6^high^ monocytes and MT1G^high^ macrophages), we reconstructed two monocyte-to-macrophage lineages using Slingshot. These consisted of a common branch of FCN1^high^ monocytes transitioning into IL1B^high^ monocytes, followed by CCL2^high^ and CCL18^high^ monocyte-derived macrophages. The latter subsequently branched into RGS1^high^ monocyte-derived macrophages (RGS1-lineage), or via FABP4^medium^ into FABP4^high^ tissue-resident macrophages (alveolar lineage; Fig. 5e). Monocyte marker expression decreased along both lineages, while macrophage marker expression increased (Fig. 5f; Supplementary information, Fig. S3d), as reported previously.^19,31,32^ Density plots revealed that in COVID-19 cells were enriched in the first half of both lineages (Fig. 5g), confirming our above observations of monocyte enrichment in COVID-19. Comparing mild to critical COVID-19 revealed a similar difference (Fig. 5h).

Modeling gene expression along the alveolar lineage revealed 5 gene sets (Fig. 5i; Supplementary information, Table S9). Sets 1 and 2 were characterized by inflammatory markers (*CXCL1-3*, *CCL20*, *CXCL8*, *CXCL10*, *CCR1*, *IL1B*), survival factors (*RAC1*, *JAK1*, *ZEB2*, *CDKN1A*), IFN-induced (anti-viral) genes (*IFITM1-3*, *IFIT1-3*, *IRF1*, *MX1/2*), hypoxia (*HIF1A*) and NF-κB (*NFKB1/2*, *NFKBIZ*) signaling early in the lineage, suggesting monocytes to be characterized by a hyperinflammatory state. The third gene set was characterized by a possible CD47-based macrophage-suppressive phenotype, potentially aimed at dysregulating macrophage-activation (since CD47 is a well-established ‘don’t eat me’ signal striving to avoid auto-immunity).^33,34^ Moreover, based on expression of purinergic signaling (*P2RX7*), inflammasome or IL1-modulating factors (*NLRP3*, *IL1B*, *IL10RA*, *CTSL*, *CALM1*, *NFKB1*), endoplasmic reticulum (ER) stress capable of enabling ATP secretion (*UBC*, *PSMB9*, *SEC61G*, *ATF5*, *ATF3*), unconventional trafficking (*VAMP5*), fibrosis-related factors (*FGL2*, *TGFB1*, *COTL1*) and vascular inflammation (*TNF*, *AIF1*, *RNF213*, *CCL2*, *CCL8*) across sets 2 and 3 of these monocytes, we strongly suspect presence of extracellular ATP-driven purinergic-inflammasome signaling; especially given the high likelihood of extracellular ATP release from damaged epithelium in the context of acute viral infection. This was confirmed by ATP measurements on BAL supernatant, showing a 3-fold higher ATP level in critical COVID-19 vs non-COVID-19 (*P* = 0.016; Fig. 5j). A similar trend was seen comparing critical vs mild COVID-19 patients, yet not reaching statistical significance due to limited sample size (*P* = 0.34). Importantly, this ATP-driven purinergic-inflammasome signaling pathway is a danger signaling cascade, which has been shown to facilitate ARDS-associated lung fibrosis and could thus act disease-worsening in this context.^35–37^ Finally, set 4 and 5 genes were expressed at the end of the trajectory. Set 4 was characterized by expression of chaperone-coding genes (*CALU*, *CALR*, *CANX*, *PDIA4, HSP90B1*), which are crucial for robust functioning of the antigen-loading machinery for MHC molecules, whereas in set 5 there were clear signs of antigen presentation (expression of numerous MHC class II genes). Furthermore, set 5 comprised genes involved in receptor-mediated phagocytosis and post-phagocytic lipid degradation/metabolism: *APOE* for lipid metabolism, scavenger receptors *MARCO* and *MSR1*, complement activation (*C1QA*, *C1QB*, *C1QC* and *CD46*; that can also facilitate phagocytosis), viral infection-relevant inflammatory orientation (*CD81*, *CD9*), as well as anti-inflammatory markers (*PPARG*, *FABP4*).^38,39^ Similar gene sets were observed for the RGS1-lineage (Supplementary information, Fig. S3e and Table S10), except for gene set 5, which exhibited expression of genes involved in chemokine signaling desensitization (*RGS1*), phagocytosis (*AXL*) and ATP clearance (*ENTPD1*).^40^

Overall, this indicates that mild COVID-19 is characterized by functional pro-phagocytic and antigen-presentation facilitating functions in myeloid cells, whereas critical COVID-19 is characterized by disease-worsening characteristics related to monocyte-based macrophage suppression and ATP-purinergic signaling-inflammasome that may enable COVID-19 associated fibrosis and can worsen patient prognosis.

### Qualitative assessment of T-cell and monocyte/macrophage function in COVID-19

Next, although pseudotime inference allocates cells with a similar expression to the same pseudotime, we explored differences in gene expression along the pseudotime. We scored each cell using REACTOME signatures and when comparing COVID-19 vs non-COVID-19 BAL, we observed consistently decreased IFN-signaling in non-COVID-19 T-cell and myeloid lineages (Supplementary information, Fig. S4a). In mild vs critical COVID-19, we observed that amongst several other pathways, IFN- (type I and II), interleukin (e.g., IL12 and IL6) and oligoadenylate synthetase (OAS) antiviral response signaling was increased in CD8^+^ T_RM_- and T_EX_-lineages (Fig. 5k; Supplementary information, Fig. S4b, e). The CD4^+^ T_H1_-lineage was similarly characterized by increased IFN- (type I and II) and interleukin (IL6, IL12, IL21) signaling in mild COVID-19 (Fig. 5l; Supplementary information, Fig. S4f, g). Additionally, TRAF6-induced NF-κB and IRF7 activation, as well as TGFBR complex activation were increased. Similar effects were observed in the T_H17_-lineage (Supplementary information, Fig. S4h, i). The alveolar macrophage lineage was characterized by increased phagocytosis-related pathways (scavenging receptors, synthesis of lipoxins or leukotrienes) and IFN-signaling in mild COVID-19 (Fig. 5m; Supplementary information, Fig. S4j–m). Vice versa, IL10-signaling (which inhibits the IFN-response), chemokine receptor binding and ATF4-mediated ER stress response were increased in critical COVID-19.

Overall, since the IFN-signaling pathway was enriched in mild vs critical COVID-19, we assessed specific expression of IFN genes and their receptors, as well as several key chemokines/cytokines related to anti-viral responses (Supplementary information, Fig. S5a). IFN genes *IFNG* and *IFNE*, and IFN receptors *IFNAR2* and *IFNGR2*, were upregulated in COVID-19 vs non-COVID19, suggesting a crucial role for IFN-signaling in mounting an anti-SARS-Cov-2 response. This also applied to *IL6*, *CCL3* and *CCL4*, as well as several other IFN response genes (*IFITM1/2*, *MX2*, *IRF7;* Supplementary information, Fig. S5c). When comparing COVID-19 mild vs critical patients, we identified *IFNLR1, IFNA1, CXCL9, CXCL10, IL15* and *IL15RA* to be upregulated in mild patients (Supplementary information, Fig. S5b). There was also significant upregulation of MHC-II expression (*HLA-DRB5*, *HLA-DQB2*, *HLA-DPB1, etc*.; Supplementary information, Fig. S5d) in mild COVID-19 patients.

Overall, while our trajectory analyses already indicated quantitative shifts in cellular phenotypes comparing mild vs critical COVID-19, we here found that qualitatively immune cells from critical COVID-19 are also severely dysfunctional. Particularly, we observed that functional IFN-signaling is one of the features distinguishing mild from critical COVID-19 patients.

### ScRNA-seq of neutrophils, DCs and B-cells in COVID-19

**Fig. 6:**
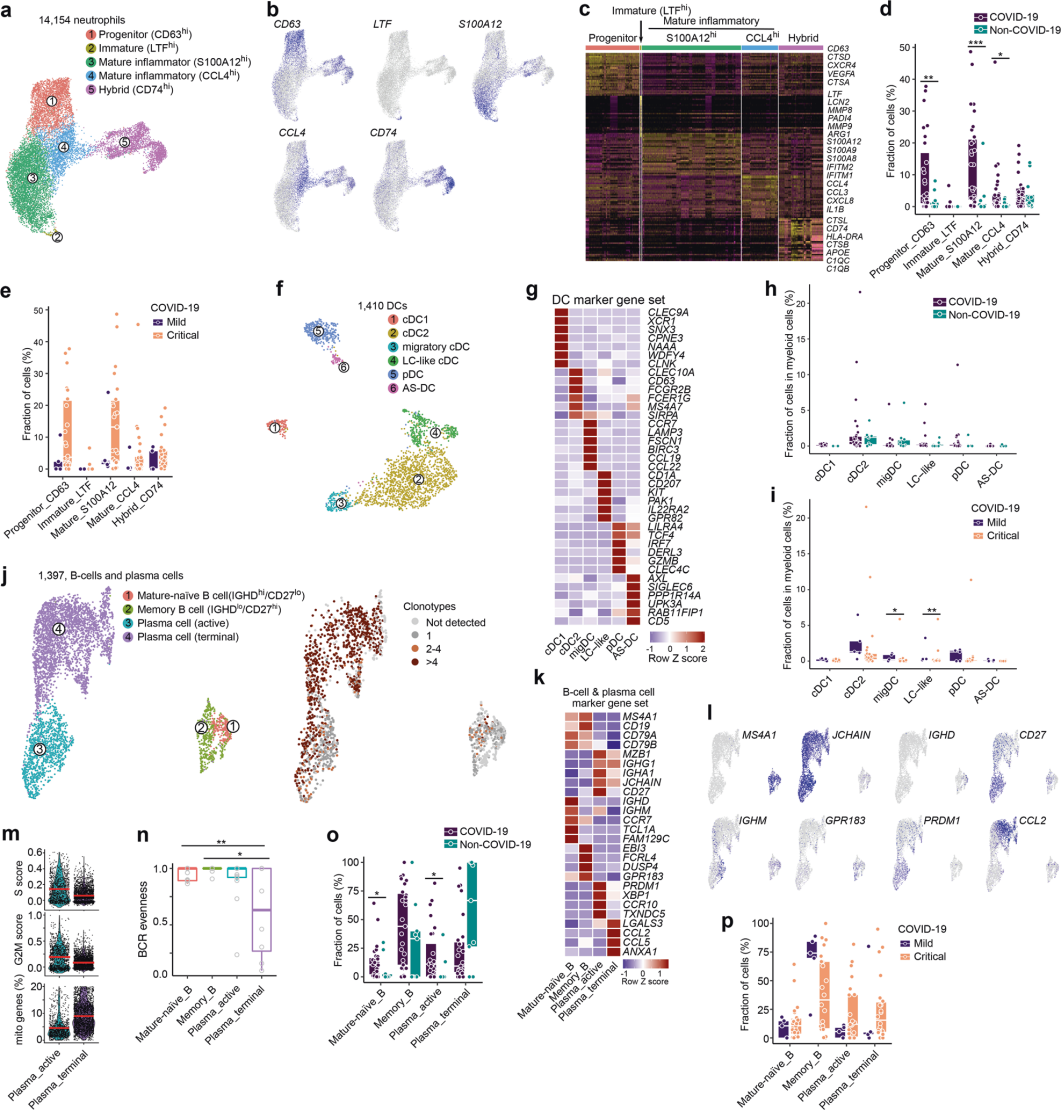
Neutrophil, dendritic cell and B-cell phenotypes in COVID-19 BAL. **a** Subclustering of neutrophils into 5 phenotypes, as indicated by the color-coded legend. **b** UMAP showing expression of a marker gene for each neutrophil phenotype. **c** Heatmap showing neutrophil phenotypes with corresponding marker genes and functional gene sets. **d** Relative contribution of each neutrophil phenotype (in %) to COVID-19 vs non-COVID-19. **e** Relative contribution of each neutrophil phenotype (in %) to mild vs critical COVID-19. **f** Subclustering of DCs into 6 phenotypes, as indicated by the color-coded legend. **g** Heatmap showing DC phenotypes with corresponding marker genes and functional gene sets. **h** Relative contribution of each DC phenotype (in %) to COVID-19 vs non-COVID-19. **i** Relative contribution of each DC phenotype (in %) to mild vs critical COVID-19. **j** Subclustering of B-cells and plasma cells into 4 phenotypes, as indicated by the color-coded legend. **k** Heatmap showing B-cell and plasma cell phenotypes with corresponding marker genes and functional gene sets. **l** Feature plots of marker gene expression for each B-cell and plasma cell subcluster. **m** Violin plots showing cell cycle scores and mitochondrial gene expression by plasma cell subcluster. **n** B-cell receptor evenness in B-cell and plasma cell subclusters. **o** Relative contribution of each B-cell and plasma cell phenotype (in %) to COVID-19 vs non-COVID-19. **p** Relative contribution of each B-cell and plasma cell phenotype (in %) to mild vs critical COVID-19. *P* values were assessed by a Mann–Whitney test. *P < 0.05, **P < 0.01, ***P < 0.001.

We used an extensive set of neutrophil and monocyte marker genes to differentiate 14,154 neutrophils from monocytes (Supplementary information, Fig. S6a, b), and compared these to published scRNA-seq datasets to confirm that they represented reliable neutrophil markers^8,14,41^ (Supplementary information, Fig. S6c–e). The neutrophils were subclustered into 5 phenotypes (Fig. 6a, b; Supplementary information, Fig. S6f). A first cluster consisted of ‘progenitor’ neutrophils based on *CXCR4* and *CD63*, and was also characterized by expression of the angiogenic factor *VEGFA* and cathepsins (*CTSA*, *CTSD*) (Fig. 6c). A second cluster consisted of few ‘immature’ neutrophils expressing *LTF*, *LCN2*, *MMP8*/9, *PADI4* and *ARG1*. Cluster 3 and 4 consisted of ‘inflammatory mature’ neutrophils, both expressing a signature footprint that highlights anti-pathogenic orientation of neutrophils:^42^ cluster 3 expressed IFN-induced genes and calgranulins (*S100A8/9*, *S100A9* and *S100A12*), which can modulate inflammation, while cluster 4 expressed high levels of cytokines (*IL1B*) and chemokines (*CXCL8, CCL3*, *CCL4*). A final subset was characterized as ‘hybrid’ neutrophils due to their macrophage-like characteristics, i.e., expression of MHC class II and complement activation genes (*C1QB*, *C1QC, CD74*), cathepsins (*CTSB*, *CTSL*) and *APOE*. All neutrophil subclusters were more frequent in COVID-19 than non-COVID-19, but most significant changes were noticed for the ‘progenitor’ and ‘inflammatory mature’ neutrophils (Fig. 6d), both for the mild and critical COVID-19 vs non-COVID-19 comparison, albeit not always significantly (Fig. 6e; Supplementary information, Fig. S6g). Indeed, the enrichment for neutrophils in COVID-19 was independent of disease severity, lung injury and bacterial infection (Supplementary information, Table S11), as well as age, gender and underlying disorders. Overall, this suggests that neutrophils play a specific role in COVID-19 pneumonia and do not just represent a marker of severe lung inflammation.

We also identified 1410 dendritic cells (DCs), which we could subcluster into 6 established populations (Fig. 6f, g; Supplementary information, Fig. S6h, i). None of these differed significantly between COVID-19 and non-COVID-19, while migratory DCs and Langerhans-cell-like DCs were more frequent in mild vs critical COVID-19 (Fig. 6h, i; Supplementary information, Fig. S6j).

Within the 1397 B-cells, we obtained 4 separate clusters (Fig. 6j; Supplementary information, Fig. S6k). Follicular B-cells were composed of ‘mature-naïve’ (CD27^–^) and ‘memory’ (CD27^+^) B-cells. The former were characterized by a unique CD27^–^/IGHD^+^(IgD)/IGHM^+^(IgM) signature and give rise to the latter by migrating through the germinal center to form CD27^+^/IGHD^–^(IgD)/IGHM^–^(IgM) memory B-cells (Fig. 6k, l). Memory B-cells then further differentiate into antibody-secreting plasma cells (*IGHA1*, *IGHG1*, *JCHAIN*), which themselves can be further classified into short-lived and long-lived plasma cells. We failed to identify long-lived plasma cells (as these are non-proliferating cells residing in bone marrow and spleen,^43,44^ but were able to divide short-lived plasma cells into two subclusters. A first subcluster of ‘active’ plasma cells expressed high levels of *PRDM1*(Blimp-1) and *XBP1*, indicating high antibody-secretion capacity, while a second cluster referred to as ‘terminal’ plasma cells was enriched for *CCL2* and *CCL5*, but also characterized by reduced G2M and S cell cycle scores and increased expression of mitochondrial genes, indicating ongoing stress (Fig. 6m). Notably, the latter population was characterized by increased BCR clonality and reduced BCR evenness (Fig. 6n). Compared to non-COVID-19, mature-naïve B-cells and active plasma cells were increased in COVID-19, while terminal plasma cells were reduced in COVID-19, albeit non-significantly (Fig. 6o). Although these effects were mainly driven by critical COVID-19 vs non-COVID-19 (Supplementary information, Fig. S6l), there were no significant differences between mild vs critical COVID-19 (Fig. 6p). Overall, this suggests terminal plasma cells in COVID-19 are characterized by sub-optimal differentiation or activation, which may cause defective or counter-productive (possibly low-quality) antibody responses in COVID-19.

### SARS-CoV-2 viral particles in epithelial and immune cells

**Fig. 7:**
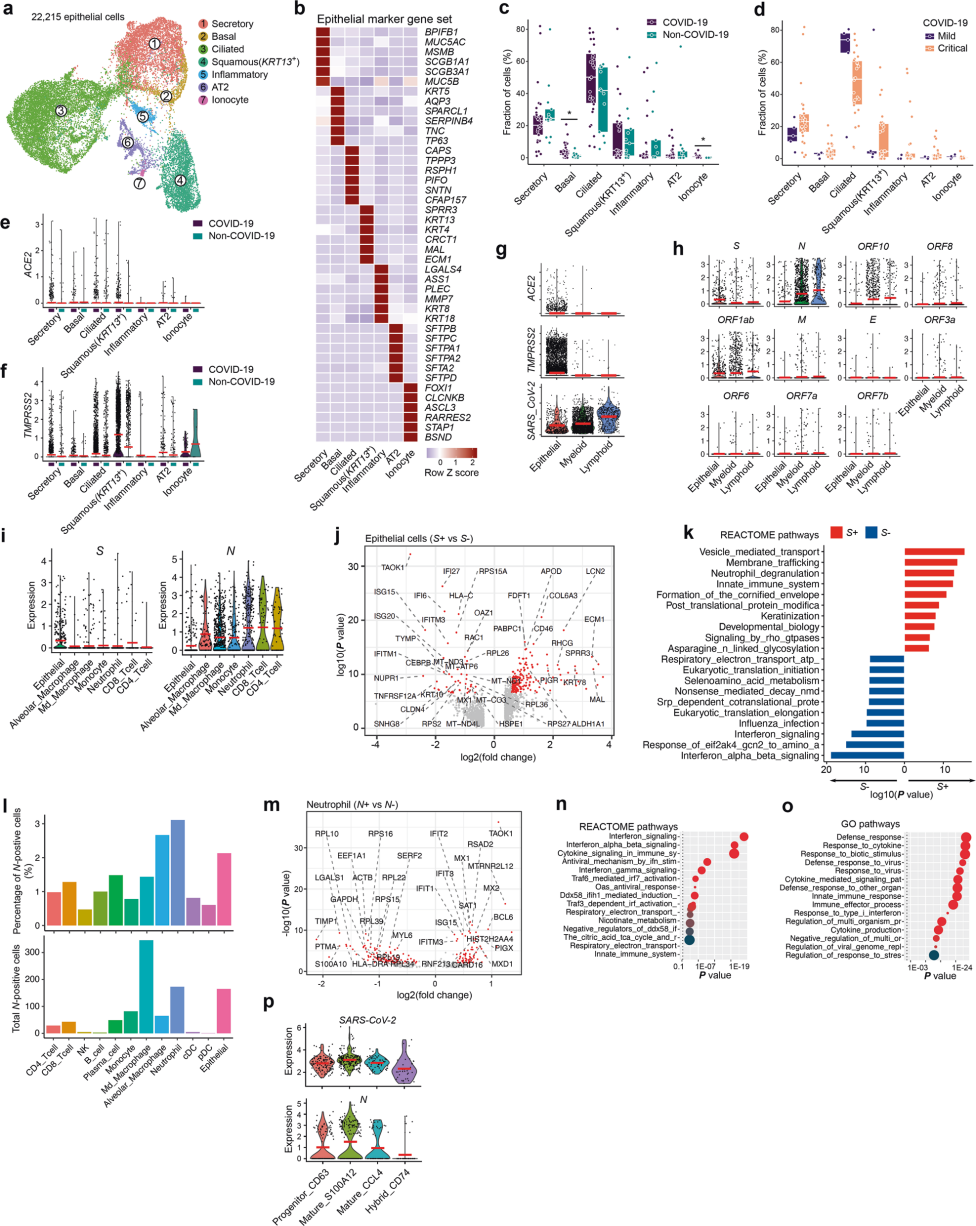
SARS-CoV-2 RNA detection in epithelial and immune cells. **a** Subclustering of epithelial cells into 7 phenotypes, as indicated by the color-coded legend. **b** Heatmap showing epithelial cell phenotypes with corresponding marker genes. **c** Relative contribution of each epithelial cell phenotype (in %) to COVID-19 vs non-COVID-19. **d** Relative contribution of each epithelial cell phenotype (in %) to mild vs critical COVID-19. **e, f** Expression level of *ACE2* (**e**) and *TMPRSS2* (**f**) by epithelial cell subclusters, comparing COVID-19 vs non-COVID-19. **g** Expression levels of *ACE2*, *TMPRSS2* and *SARS-CoV-2 (*cells with viral reads) RNA in epithelial, myeloid and lymphoid cells from COVID-19. **h** Detection of 11 SARS-CoV-2 open-reading frames in epithelial, myeloid and lymphoid cells from COVID-19. **i** Detection of spike protein (*S*) and nucleocapsid protein (*N*) encoding viral RNA in epithelial cells and immune cell subclusters from COVID-19. Cell types with < 50 positive cells are not shown. **j** Differential gene expression of *S*^*+*^ vs *S*^–^ epithelial cells from 17 COVID-19 patients in which viral reads were detected. **k** REACTOME pathway analysis based on differentially expressed genes between *S*^*+*^ vs *S*^*-*^ virus infected epithelial cells. **l** Relative percentage of cells containing reads mapping to the viral *N* gene (upper panel) and total number of cells that contain reads mapping to the *N* gene (lower panel) stratified for each of the cell types. **m** Differential gene expression of *N*^+^ vs *N*^–^ neutrophils from 17 COVID-19 patients in which viral reads were detected. **n, o** REACTOME (**n**) and GO (**o**) pathway analysis on IFN-signaling and response-to-virus signaling, comparing *N*^+^ vs *N*^–^ neutrophils from 17 COVID-19 patients in which viral reads were detected. **p** Detection of reads mapping to *SARS-CoV-2* and to *N* in neutrophil subclusters from COVID-19 BAL. *P* values were assessed by a Mann–Whitney test. *P < 0.05, **P < 0.01, ***P < 0.001.

Finally, we retrieved 22,215 epithelial cells, which we subclustered similar to Chua et al. into 7 distinct clusters (Fig. 7a, b; Supplementary information, Fig. S7a–e), the largest 3 clusters consisting of secretory, ciliated and squamous lung epithelial cells.^16,45^ Although epithelial cells were less frequent in COVID-19 vs non-COVID-19 patients, basal cells, representing stem cell epithelial cells responsible for epithelial remodelling upon lung injury, were significantly enriched in COVID-19, as well as ionocytes, a rare epithelial cell type that regulates salt balance (Fig. 7c; Supplementary information, Fig. S7f). There were no significant differences between mild vs critical COVID-19 (Fig. 7d). Interestingly, *ACE2* and *TMPRSS2* expression was increased in COVID-19 vs non-COVID-19, with respectively 2.3% and 21% of COVID-19 epithelial cells expressing these genes (Fig. 7e, f). We then assessed in which cells we retrieved sequencing reads mapping to the SARS-CoV-2 genome, identifying 3773 positive cells from 17 out of 31 COVID-19 patients. Surprisingly, this revealed a higher overall number of reads mapping to lymphoid and myeloid than epithelial cells (Fig. 7g). Stratification for each of the 11 SARS-CoV-2 open-reading frames (ORF) using Viral-Track revealed that the RNA encoding for spike protein (*S*), which interacts with ACE2 during viral entry of the cell, was almost exclusively detected in epithelial cells, which were also the only cells expressing *ACE2* and *TMPRSS2* (Fig. 7h, i). Intriguingly, differential gene expression analysis revealed that *S*^*+*^-epithelial cells exhibited reduced expression of IFN-stimulated genes (*IFI6/27*, *ISG15*/*20*, *IFITM1*/*3 etc*.; Fig. 7j) and IFN-signaling pathways (Fig. 7k) relative to *S*^*-*^-epithelial cells. Indeed, viruses must overcome IFN-mediated antiviral response in order to replicate and propagate,^46^ suggesting that *S*^*+*^-epithelial cells have actively been infected. In contrast, the nucleocapsid protein (*N*), and to a lesser extent the ORF10 and ORF1a-encoding mRNAs were detected in myeloid and lymphoid cells at much higher levels than in epithelial cells (Fig. 7h). Further stratification into cell types, relative to the number of cells present in BAL, revealed that neutrophils and macrophages were the most frequent *N*^*+*^-cell type (Fig. 7l). Differential gene expression of *N*^+^- vs *N*^–^-neutrophils identified upregulation of transcription factor *BCL6*, which promotes neutrophil survival and inflammatory response following virus infection, and numerous IFN-induced genes (*IFITM3*, *IFIT1-3*, *MX1/2*, *ISG15*, *RSAD2, etc*.; Fig. 7m).^47^ When comparing *N*^*+*^- vs *N*^*-*^*-*alveolar and -mono-derived macrophages, we noticed that genes involved in MHC class-II expression and to some extent also IFN-induced genes (*IFIT3*, *IRF7*, *MX2, OAS1, OAS3, etc*.) were overexpressed, an effect that was less obvious in *N*^*+*^- vs *N*^−^-monocytes (Supplementary information, Fig. S7g–i). Pathway analysis on differentially-expressed genes revealed IFN-signaling using REACTOME and Response_to_virus using GO for genes upregulated in *N*^*+*^-neutrophils (Fig. 7n, o). Notably, amongst the different neutrophil phenotypes, *N* was most strongly enriched in ‘inflammatory mature’ neutrophils expressing calgranulins (Fig. 7p). As expected, significantly more *N* was present in critical vs mild COVID-19 (Supplementary information, Fig. S7j, k).^48^

Overall, while *S*^*+*^-(infected) epithelial cells reduce IFN-signaling, *N*^*+*^-neutrophils are characterized by increased IFN-signaling, suggesting neutrophils to be the main cell type interacting with SARS-CoV-2 viral particles and to account for the highest procurement of viral material, in line with their role as first innate immune responders to infection.^49^

### Cell-to-cell communication to unravel the immune context of COVID-19 BAL

**Fig. 8:**
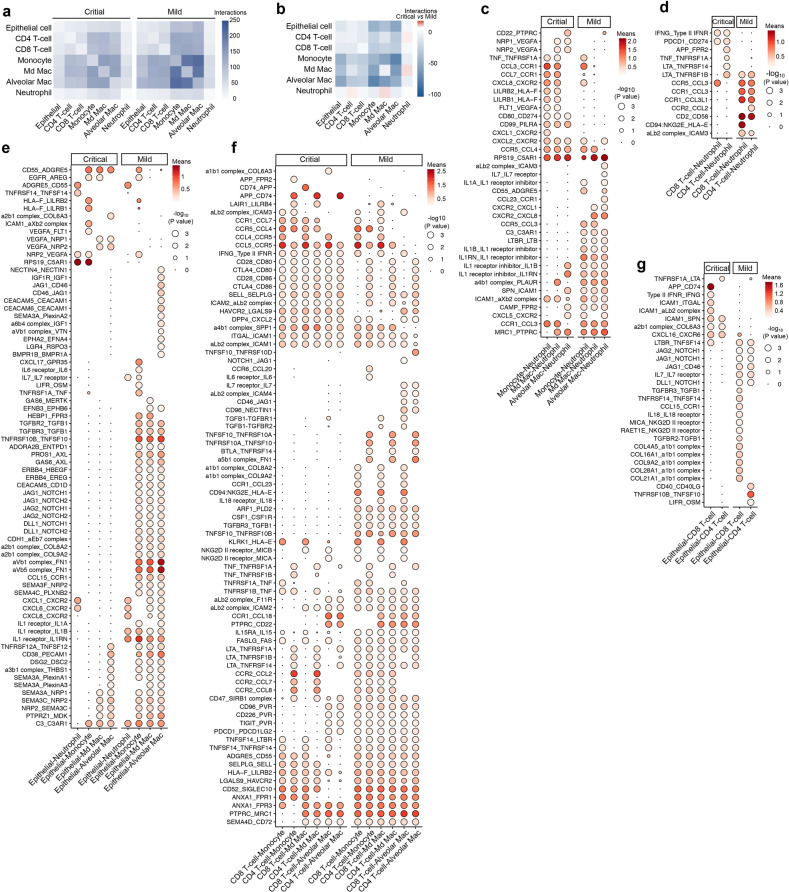
Cell-to-cell communication between epithelial and immune cells. **a** Number of predicted interactions (*P* ≤ 0.05) between monocytes, macrophages, T-cells, neutrophils and epithelial cells based on CellPhoneDB in critical (left panel) and mild (right panel) COVID-19. **b** Differences in the number of predicted interactions, comparing mild vs critical COVID-19, showing generally more interactions in mild COVID-19. **c** Predicted interactions between monocytes/macrophages and neutrophils, comparing critical vs mild COVID-19. **d** Predicted interactions between T-cells and neutrophils, comparing critical vs mild COVID-19. **e** Predicted interactions between epithelial and myeloid cells, comparing critical vs mild COVID-19. **f** Predicted interactions between T-cells and monocytes/macrophages, comparing critical vs mild COVID-19. **g** Predicted interactions between T-cells and epithelial cells, comparing critical vs mild COVID-19.

Since, our data on the one hand reveal that neutrophils were involved in cleaning up viral particles/virus-infected cells, yet T-cell and monocyte-to-macrophage lineages were significantly disrupted in critical COVID-19, we explored the (predicted) interactome between these cell types to gain more refined insights. First, we calculated interactions between cell types (*P* ≤ 0.05) separately for mild and critical COVID-19, then we assessed differences in the number of specific interactions. Neutrophils were characterized by a low number of specific interactions that were slightly more frequent in critical vs mild COVID-19. Vice versa, numerous specific interactions were predicted between all other immune and epithelial cells, especially in mild COVID-19 (Fig. 8a, b; Supplementary information, Fig. S8).

In critical COVID-19, specific interactions between monocytes/macrophages and neutrophils almost always involved pro-migratory interactions (*FLT1*, *NRP1* or *NRP2*/*VEGFA*, *CXCL1* or *CXCL2* or *CXCL8*/*CXCR2*, *CCL3* or *CCL7*/*CCR1*), coupled with immune-inhibitory interactions, such as *LILRB1* or *LILRB2*/*HLA-F* and *RPS19*/*C5AR1*, which also induce neutrophil dysfunction (Fig. 8c).^50^ A few stimulatory T-cell to neutrophil interactions were observed, including *IFNG*/*type II IFNR*, *PDCD1*/*CD274*, *LTA*/*TNFRSF1A* or *TNFRSF1B* (Fig. 8d), while specific epithelial cell-to-neutrophil interactions were limited to a mixture of myeloid immunosuppression (*RPS19*/*C5AR1*) and viral infection-relevant pro-inflammatory signals (*TNFRSF14*/*TNFSF14*) (Fig. 8e). Amongst T-cell and monocytes/macrophages, some immune-stimulatory or auto-regulatory interactions were seen (*CTLA4* or *CD28*/*CD80* or *CD86*, *CCL5/CCR5*) (Fig. 8f), but specific epithelial to T-cell interactions in critical COVID-19 were limited to pro-inflammatory ICAM1-mediated interactions (Fig. 8g).

A very different scenario was observed in mild COVID-19 (Fig. 8c–g). Amongst the numerous interactions between monocytes/macrophages and neutrophils, we noticed interleukin signaling (bi-directional *IL1B*, *IL1A, IL1RN/A* signaling, *IL7*/*IL7R*, *CXCR2*/*CXCL1* or *CXCL8*), but also *MRC1*/*PTPR* (phagocytosis) and *LTBR/LTB* (pro-inflammation). Between T-cells and neutrophils specific interactions involved *CCR1*/*CCL3* or *CCL3L1* (pro-inflammation), *CD2*/*CD58* (co-stimulatory/immunogenic pathway) and *CD94:NKG2E*/*HLA-F* (anti-viral immune-surveillance), whereas between epithelial cells and neutrophils, *IL1R*/*IL1A* or *IL1B* or *IL1R* interactions were most pronounced (which can facilitate productive neutrophil immunity in an immune-controlled/immunogenic context).^42,51,52^ Numerous interactions were also observed between epithelial cells and monocyte/macrophages: *GAS6* or *PROS1*/*AXL* (receptor-mediated phagocytosis), *ADORA2B*/*ENTPD1* (extracellular ATP degradation/suppression), *CD83*/*PECAM1* (immune activation) and semaphorins interacting with their plexin and NRP receptors (tissue re-modelling and repair). Between epithelial cells and T-cells, we observed mainly co-stimulatory (*CD46*/*JAG1*, *CD40LG*/*CD40*, *IL7R*/*IL7*, *MICA* or *RAET11/NKG2D receptor*) and tissue repair interactions (*TGFB1/TGFR2* and *TGFB1/TGFBR3)*, while amongst T-cells and monocytes/macrophages, there were amongst others, co-stimulatory (*LTA*/*TNFRSF1A* or *TNFRSF1B* or *TNFRSF14*, *TNFSF10*/*TNFRSF10B*) or tissue-repair factors (*CSF1*/*CSFR1*, *TGFBR3*/*TGFB1*, *IL15RA*/*IL15*), mediators of T-cell homeostasis and cytotoxicity (*FASLG*/*FAS*) and antiviral immune surveillance (*NKG2D II receptor*/*MICB* or *MICA*).

## Discussion

Using scRNA-seq data derived from BAL, we performed deep-immune profiling of the adaptive and innate immune cell landscape within the main locale of COVID-19 pathology. We analyzed a fairly large number of COVID-19 patients (*n* = 31), enabling statistically meaningful and robust comparisons between mild and critical disease groups (in contrast to initial COVID-19 publications profiling < 10 patients).^14^ Due to the fact that we profiled > 116,000 single-cells, we could infer pseudotime trajectories for both T-cells and myeloid cells. Such method is particularly attractive since it allows modelling of gene expression changes along the inferred trajectories, thereby generating data at a much greater resolution. Overall, we could draw the following key conclusions regarding what distinguishes a critical from a mild COVID-19 disease course:

Firstly, CD8^+^ T-cells exhibited good effector functions along their resident-memory and partially-exhausted lineages in mild COVID-19, while also CD4^+^ T-cells showed disease-resolving functions in T_H1_- and T_H17_-lineages. In critical COVID-19, T-cells were highly dysregulated, either getting stuck in a naïve state (T_H17_- and T_RM_-lineage) or becoming severely dysregulated due to inflammation-associated stress (T_EX_- and T_H1_-), thereby leading to metabolic disparities, proteotoxic stress and chromatin re-modelling, as well as dysregulation of their immunological interface with myeloid cells. Interestingly, we observed that mild vs critical COVID-19, not only differed quantitatively in terms of the number of T-cells exhibiting good effector function, but also qualitatively, in terms of consistently lower activation levels of the type 1 and II IFN (anti-viral) signaling pathways (amongst several other pathways). Overall, this showed that T-cells in mild COVID-19, unlike those in critical COVID-19, were cross-talking better with their lung-localized microenvironment thereby facilitating ‘ordered’ immune reactions capable of resolving, rather than exacerbating, inflammation and tissue repair.^53^

Secondly, in mild COVID-19 macrophages characterized by anti-inflammatory, pro-phagocytic and antigen-presentation facilitating functions were enriched. This suggests that in these patients, macrophages might be cleaning the dying/dead epithelial cells (as well as other immune cells meeting their demise due to inflammation), hence contributing to degradation and dilution of the viral load in COVID-19 BAL. Such pro-homeostatic activity of macrophages is well-established to aid in disease amelioration and inflammation resolution.^54^ In critical COVID-19, monocytes were much more frequent and characterized by a chronically hyper-inflamed phenotype, as reported previously.^14,16^ Importantly, these pro-inflammatory monocytes exhibited an ATP-purinergic signaling-inflammasome, which can promote worse disease outcome by contributing to development of ARDS and fibrosis. In vitro ATP measurements on BAL supernatant confirmed increased ATP levels in COVID-19 BAL. Considering that fully-differentiated macrophages are much more efficient in clearing large debris or cellular corpses (e.g., infected dead/dying lung epithelia or dead neutrophils) than monocytes or neutrophils, their dysfunction in critical COVID-19 may explain the excessive accumulation of lung epithelial (as well as dead immune cell) debris and alveolar dyshomeostasis coupled with dysregulated coagulopathy.^55–59^

Lastly, based on the presence of sequencing reads mapping to the gene encoding for viral protein S, which is needed to infect cells via ACE2 and TMPRSS2 receptors, we propose that SARS-CoV-2 infects epithelial cells (as primary targets of excessive pathological replication and propagation), but not necessarily lymphoid or myeloid cells (although we cannot exclude that some virions might be capable of ‘latently’ entering these cells without showing pathological replication or propagation). Interestingly, *S*^*+*^-epithelial cells were ‘suppressed’ for IFN-signaling, indicating a viral replication permissive state.^46^ We also detected reads mapping to the nucleocapsid protein (*N*) encoding gene mainly in neutrophils and macrophages. In contrast to *S*^*+*^-epithelial cells, *N*^+^-neutrophils (and to a lesser extent also macrophages) exhibited ‘increased’ expression of IFN-induced (anti-viral) genes relative to *N*^*-*^-neutrophils. This suggests that neutrophils might be heavily involved in viral clearance of SARS-CoV-2 – as is the case in most viral pathologies,^49^ while macrophages are more dedicated towards clearing the cellular debris. Some sequencing reads also mapped to *ORF10* and *ORF1ab*, but not the other viral protein-encoding genes. We suspect this is due to increased stability of *N*, *ORF10* and *ORF1ab* RNA compared to other viral ORFs.

Importantly, we also profiled BAL from non-COVID-19 pneumonia cases (*n* = 13), instead of healthy controls. Since the latter are likely to differ on almost every immunological parameter relative to COVID-19, our strategy of including non-COVID-19 pneumonia as a control enhances qualitative clarity of our immunological conclusions. Particularly, in comparison to non-COVID-19, we found that monocytes from COVID-19 patients were enriched in critical, but not in mild disease. Neutrophils were also enriched in COVID-19, but their number did not correlate with disease severity. On the other hand, although macrophages were increased in mild vs critical COVID-19, their relative numbers were similar when comparing COVID-19 vs non-COVID-19, both in the mild and critical disease comparisons. Finally, T-cells were increased in mild COVID-19 vs non-COVID-19, but not in critical patients where they were much less frequent. Overall, these numbers support a model wherein neutrophils execute their antiviral function in an immunologically ‘controlled’ fashion, regulated by T-cells with good effector functions and paralleled by ‘orderly’ phagocytic disposal of expired cells by macrophages in mild disease. In contrast, in critical disease, T-cells are less abundant and dysregulated, which coupled to hyperinflammatory monocytes facilitates excessive neutrophil-based inflammation.

These findings bear important therapeutic relevance. The RECOVERY trial recently claimed that dexamethasone reduces death by one-third in hospitalized patients with critical COVID-19.^60^ Dexamethasone has indeed been shown to dampen myeloid inflammatory signaling (notably IL-1 and IL-6 release), reduce neutrophil inflammation,^61^ promote a macrophage phenotype with anti-inflammatory and phagocytic traits,^62^ and to maintain clonal balance in T-cells.^63^ Multiple other drugs known to shift the macrophage phenotype towards the anti-inflammatory ‘M2-like’ spectrum, e.g., infliximab (NCT04425538) or decitabine (NCT04482621), are actively being evaluated in the context of (severe) COVID-19. Our data also suggest that neutrophils are key players in the acute phase of the infection. However, prolonged neutrophil inflammation might also cause excessive collateral lung damage and be detrimental to the host, as suggested by autopsy reports.^64^ In this regard, it remains to be seen whether the immunomodulatory antibiotic azithromycin, which modulates neutrophil function, represents a promising therapy for COVID-19.

Nevertheless, there are also limitations to our study. For instance, we observed evidence of counter-productive (possibly low-quality) antibody response-related signatures in COVID-19, but failed to characterize this in full-depth. Additional studies performing scRNA- and scBCR-seq on serially-collected samples during disease are needed to reinforce this observation. Also, several COVID-19 patients were treated with the antiviral drugs remdesivir, which targets the viral RNA-dependent RNA polymerase, or hydroxychloroquine, which has immunomodulatory traits (but no clinically relevant anti-viral effects).^65–67^ Of note, we did not detect major patient-specific cell clusters nor other type of outliers during our analyses. Finally, it should be stressed that when interpreting data derived from trajectory analyses, it is important to realize that cells originally derived from a tissue but now residing elsewhere will be missed in the trajectory analysis, while cells that have infiltrated this tissue, but are unrelated to the other cells residing in the tissue (e.g., peripheral bystander T-cells or alveolar macrophages homing into the lung), will be analyzed as if they are part of the trajectory. Trajectory analyses should therefore not be interpreted as actual differentiation experiments.

In conclusion, we used single-cell transcriptomics to characterize the innate and adaptive lung immune response to SARS-CoV-2. We observed marked changes in the immune cell compositions, phenotypes as well as immune cross-talks during SARS-CoV-2 infection and identified several distinguishing immunological features of mild vs critical COVID-19. We also documented genetic footprints of several immunological pathways that have been extensively hypothesized, but not always systematically confirmed, to be associated with COVID-19 pathology and SARS-CoV-2 infection biology. We believe that this work represents a major resource for understanding lung-localized immunity during COVID-19 and holds great promise for the study of COVID-19 immunology, immune-monitoring of COVID-19 patients and relevant therapeutic development.

## Materials and methods

### Patient cohort, sampling and data collection

22 COVID-19 patients and 13 non-COVID-19 pneumonia patients in this study were enrolled from the University Hospitals Leuven, between March 31st 2020 and May 4th 2020. Disease severity was defined as ‘mild’ or ‘critical’, based on the level of respiratory support at the time of sampling. Specifically, ‘mild’ patients required no respiratory support or supplemental oxygen through a nasal cannula, whereas ‘critically ill’ patients were mechanically ventilated or received extracorporeal membrane oxygenation.

The demographic and disease characteristics of the prospectively recruited patients studied by scRNA-seq are listed in Supplementary information, Table S1. Diagnosis of COVID-19 was based on clinical symptoms, chest imaging and SARS-CoV-2 RNA-positive testing (qRT-PCR) on a nasopharyngeal swab and/or BAL fluid sample. Non-COVID-19 pneumonia cases all tested negative for SARS-CoV-2 RNA using a qRT-PCR assay on BAL.

All 35 patients underwent bronchoscopy with BAL as part of the standard of medical care, because of (i) high clinical suspicion of COVID-19 yet negative SARS-CoV-2 qRT-PCR on nasopharyngeal swab (ii) established COVID-19 with clinical deterioration, to rule out opportunistic (co-)infection and/or to remove mucus plugs. Lavage was performed instilling 20 mL of sterile saline, with an approximate retrieval of 10 mL. 2–3 mL of the retrieved volume was used for clinical purposes. The remaining fraction was used for scRNA-seq.

The retrieved BAL volume was separated into two aliquots, as explained above, by the performing endoscopist. The aliquot used for scRNA-seq was immediately put on ice and transported to a Biosafety Level 3 Laboratory (REGA Institute, KU Leuven) for scRNA-seq.

Demographic, clinical, treatment and outcome data from patient electronic medical records were obtained through a standardized research form in Research Electronic Data Capture Software (REDCAP, Vanderbilt University). A CT score from each patient was calculated by converting the percentage of lung parenchyma opacity for each lobe into a 5-points Likert scale (a score of 0 for 0% lung opacity (LO), 1 for 1% to < 5% LO, 2 for 5%–25% LO, 3 for 26%–50% LO, 4 for 51%–75% LO, and 5 for 76%–100% LO). The total CT score was the sum of the individual lobular scores and ranged from 0 (no area with increase in lung opacity) to 25 (all five lobes show more than 75% increase in lung opacity).

This study was conducted according to the principles expressed in the Declaration of Helsinki. Ethical approval was obtained from the Research Ethics Committee of KU / UZ Leuven (S63881). All participants provided written informed consent for sample collection and subsequent analyses.

### scRNA-seq, scTCR-seq and scBCR-seq profiling

BAL fluid was centrifuged and the supernatant was frozen at −80 °C for further experiments. The cellular fraction was resuspended in ice-cold PBS and samples were filtered using a 40 μm nylon mesh (ThermoFisher Scientific). Following centrifugation, the supernatant was decanted and discarded, and the cell pellet was resuspended in red blood cell lysis buffer. Following a 5 min incubation at room temperature, samples were centrifuged and resuspended in PBS containing UltraPure BSA (AM2616, ThermoFisher Scientific) and filtered over Flowmi 40 μm cell strainers (VWR) using wide-bore 1 mL low-retention filter tips (Mettler-Toledo). Next, 10 μL of this cell suspension was counted using an automated cell counter to determine the concentration of live cells. The entire procedure was completed in < 1.5 h.

Single-cell TCR/BCR and 5′ gene expression sequencing data for the same set of cells were obtained from the single-cell suspension using the Chromium^TM^ Single Cell 5′ library and Gel Bead & Multiplex Kit with the Single Cell V(D)J Solution from 10× Genomics according to the manufacturer’s instructions. Up to 5000 cells were loaded on a 10× Genomics cartridge for each sample. Cell-barcoded 5′ gene expression libraries were sequenced on an Illumina NovaSeq6000, and mapped to the GRCh38 human reference genome using CellRanger (10× Genomics, v3.1). V(D)J enriched libraries were sequenced on an Illumina HiSeq4000 and TCR and BCR alignment and annotation was achieved with CellRanger VDJ (10× Genomics, v3.1).

### Single-cell gene expression analysis

Raw gene expression matrices generated per sample were merged and analyzed with the Seurat package (v3.1.4).^68^ Cell matrices were filtered by removing cell barcodes with < 301 UMIs, < 151 expressed genes or > 20% of reads mapping to mitochondrial RNA. We opted for a lenient filtering strategy to preserve the neutrophils, which are transcriptionally less active (lower transcripts and genes detected). The remaining cells were normalized and the 3000 most variable genes were selected to perform a PCA analysis after regression for confounding factors: number of UMIs, percentage of mitochondrial RNA, patient ID and cell cycle (S and G2M phase scores calculated by the CellCycleScoring function in Seurat), interferon response (BROWNE_INTERFERON_RE- SPONSIVE_GENES in the Molecular Signatures Database or MSigDB v6.2), sample dissociation-induced stress signatures,^69^ hypoxia signature.^70^ Regressing out the cell cycle genes was particularly important for the T-/NK-cell subclustering. Indeed, when this was not performed a proliferating T-cell cluster that contained a mixture of different T-cell phenotypes, including CD4^+^ and CD8^+^ T-cells was identified (Supplementary information, Fig. S9a, b). The PCA and graph-based clustering approach however resulted in some highly patient specific clusters, which prompted us to perform data integration using anchor-based CCA in Seurat (v3) package between patients to reduce the patient-specific bias. And this was performed after excluding cells from an erythrocyte cluster (primarily from a single patient) and a low-quality cell cluster. After data integration, 3000 most variable genes were calculated by FindVariableFeatures function, and all the mitochondrial, cell cycle, hypoxia, stress and interferon response genes (Pearson correlation coefficient > 0.1 against scores of the above-mentioned signatures calculated by AddModuleScore function in Seurat) were removed from the variable genes. In addition, we also removed common ambient RNA contaminant genes, including hemoglobin and immunoglobulin genes, as well as T-cell receptor (TRAVs, TRBVs, TRDVs, TRGVs) and B-cell receptor (IGLVs, IGKVs, IGHVs) genes, before downstream analyses.

### scRNA-seq clustering for cell type identification

For the clustering of all cell types, principal component analysis (PCA) was applied to the variable genes of dataset to reduce dimensionality. The selection of principal components was based on elbow and Jackstraw plots (usually 25–30). Clusters were calculated by the FindClusters function with a resolution between 0.2 and 2, and visualized using the Uniform Manifold Approximation and Projection for Dimension Reduction (UMAP) reduction. Differential gene-expression analysis was performed for clusters generated at various resolutions by both the Wilcoxon rank sum test and Model-based Analysis of Single-cell Transcriptomics (MAST) using the FindMarkers function.^68^ A specific resolution was selected when known cell types were identified as a cluster at a given resolution, but not at a lower resolution with the minimal constraint that each cluster has at least 10 significantly differentially expressed genes (FDR < 0.01, twofold difference in expression compared to all other clusters). Annotation of the resulting clusters to cell types was based on the expression of marker genes.

### Integration of publicly available datasets and identification of cell subtypes

We additionally processed scRNA-seq data on COVID-19 BAL fluid by Liao et al. and on normal lung samples by Reyfman et al. and Lambrechts et al. as described above.^14,17,18^ The former two datasets were de novo clustered and annotated, and cell type annotation of the last dataset was used as previously described.^19^ For cell subtype identification, the main cell types identified from multiple datasets were pooled, integrated, and further subclustered using the similar strategy, except that the constant immunoglobulin genes were not excluded for B-cell and plasma cell subclustering. Finally, doublet clusters were identified based on: (1) expression of marker genes from other cell (sub)clusters, (2) higher average UMIs as compared to other (subclusters), and (3) a higher than expected doublets rate (> 20%), as predicted by both DoubletFinder (v2)^71^ and Scrublet^72^ and the clustering was re-performed in the absence of the doublet clusters.

### Trajectory inference analysis

The R package Slingshot was used to explore pseudotime trajectories/potential lineages in T- and myeloid cells.^73^ The analyses were performed for CD8^+^ and CD4^+^ cell phenotypes separately, with T_MAIT_-, T_γδ_- and T_REG_-cells excluded due to their unique developmental origin. For each analysis, PCA-based dimension reduction was performed with differentially expressed genes of each phenotype, followed by two-dimensional visualization with UMAP. Graph-based clustering (Louvain) identified additional heterogeneity for some phenotypes, as described in the manuscript for CD4^+^ T-cells. Next, this UMAP matrix was fed into SlingShot, with naïve T-cells as a root state for calculation of lineages and pseudotime. Similar approach was applied to the monocyte-macrophage differentiation trajectory inferences.

### Assessing the TCR and BCR repertoires

We only considered productive TCR/BCRs, which were assigned by the CellRanger VDJ pipeline. Relative clonotype richness,^74^ defined as the number of unique TCRs/BCRs divided by the total number of cells with a unique TCR/BCR, was calculated to assess clonotype diversity. Relative clonotype evenness,^75^ was defined as inverse Simpson index divided by species richness (number of unique clonotypes). To visualize the degree of TCR clonotypes shared between T-cell phenotypes, the connection weight for each pair of T-cell phenotypes was calculated as the shared number of unique TCRs divided by the total number of unique TCRs in the T-cell phenotype being located first on the trajectory. The resulting network of relatively shared TCRs was plotted using igraph packages (v1.2.5).

### Inflammatory pathways and gene set enrichment analysis and tradeSeq

The REACTOME pathway activity of individual cells was calculated by AUCell package (v1.2.4).^76^ And the differential activity between lineages along the trajectories were calculated using TradeSeq.^77^ Pathways with median fold change > 3 and an adjusted *P* value < 0.01 were considered as significantly changed. GO and REACTOME gene set enrichment analysis were performed using hypeR package;^78^ geneset over-representation was determined by hypergeometric test.

### SARS-CoV-2 viral sequence detection

Viral-Track was used to detect SARS-CoV-2 reads from BAL scRNA-seq data (reference genome NC_045512.2), as previously described.^15^ The initial application was aimed to identify SARS-CoV-2 reads against thousands of other viruses, and thus the STAR indexes for read alignment were built by combining the human (GRCh38) genome reference with thousands of virus refence genomes from viruSITE. Since the likelihood of co-infection with multiple viruses (> 2) is low in COVID-19 patients,^15^ we adapted the Viral-Track pipeline to reduce computation time and increase sensitivity. Briefly, instead of directly processing raw fastq reads, we took advantage of BAM reads generated for scRNA-seq data, which mapped to human genome by the CellRanger pipeline as described above. The BAM files were filtered to only keep reads with cell barcodes annotated in the scRNA-seq analysis using subset-bam tools (10× Genomics). Then the corresponding unmapped BAM reads were extracted using samtools and converted to fastq files using bamtofastq tool to be further processed by UMI-tools for cell barcode assignment before feeding into Viral-Track pipeline. These unmapped reads, which contain potential viral sequences, were aligned using STAR to SARS-CoV-2 reference genome, with less stringent mapping parameter (outFilterMatchNmin 25–30), as compared to the original Virial-Track pipeline. Our approach identified 17 SARS-CoV-2 positive patients from a total of 31 COVID-19 patients, including 3 patients that were previously not detected using original Viral-Track pipeline by Bost et al. None of the patients among the 13 non-COVID-19 patients were detected as SARS-CoV-2 positive, suggesting our adapted pipeline does not result in major false-positive detection. For the detection of 11 SARS-CoV-2 ORFs or genes, a GTF annotation file was generated according to NC_045512.2^79^ for counts matrix using Viral-Track. The viral gene counts of each barcoded cells were integrated into the scRNA-seq gene count matrix and normalized together using NormalizeData function in Seurat.

### Cell-to-cell communication of scRNA-seq data

The CellPhoneDB algorithm was used to infer cell-to-cell interactions.^80^ Briefly, the algorithm allows to detect ligand-receptor interactions between cell types in scRNA-seq data. We assessed the amount of interactions that are shared and specific for (i) COVID-19 vs non-COVID-19 and (ii) mild vs critical COVID-19.

### ATP measurement

50 μL of BAL supernatant was thawed and ATP was measured using the PerkinElmer^®^ ATPlite Luminescence Assay System, following manufacturer’s instructions. Measurements were performed in 3 separate batches. To account for batch effects, we calculated a normalized ATP value by dividing the measured luminescence per BAL sample (expressed in Relative Lights Units (RLU)) by the luminescence measured in the negative control sample for each batch.

### Quantification and statistical analysis

Descriptive statistics are presented as median [interquartile range; IQR] (or median [range] if dataset contained only 2 variables) and *n* (%) for continuous and categorical variables, respectively. Statistical analyses were performed using R (version 3.6.3, R Foundation for Statistical Computing, R Core Team, Vienna, Austria). Statistical analyses were performed with a two-sided alternative hypothesis at the 5% significance level.

## Supplementary information

Supplementary Figure S1

Supplementary Figure S2

Supplementary Figure S3

Supplementary Figure S4

Supplementary Figure S5

Supplementary Figure S6

Supplementary Figure S7

Supplementary Figure S8

Supplementary Figure S9

Supplementary Table S1

Supplementary Table S2

Supplementary Table S3

Supplementary Table S4

Supplementary Table S5

Supplementary Table S6

Supplementary Table S7

Supplementary Table S8

Supplementary Table S9

Supplementary Table S10

Supplementary Table S11

## Data Availability

Raw sequencing reads of the scRNA-seq and scTCR-seq experiments generated for this study have been deposited in the EGA European Genome-Phenome Archive database (EGAS00001004717). A download of the read count matrix will be made available upon publication at http://covid19.lambrechtslab.org. The publicly available datasets that supported this study are available from GEO GSE145926,^14^ GEO GSE122960^18^ and from ArrayExpress E-MTAB-6149/E-MTAB-6653.^17^
