# Supplementary material for: Discriminating mild from critical COVID-19 by innate and adaptive immune single-cell profiling of bronchoalveolar lavages
Source: Cell Res. 2021 Jan 21;31(3):272–90. doi: 10.1038/s41422-020-00455-9 (PMC8027624; doi:10.1038/s41422-020-00455-9)
Supplement: Supplementary file 1 — Supplementary Figure S1 [file 41422_2020_455_MOESM1_ESM.pdf]

Fig. S1

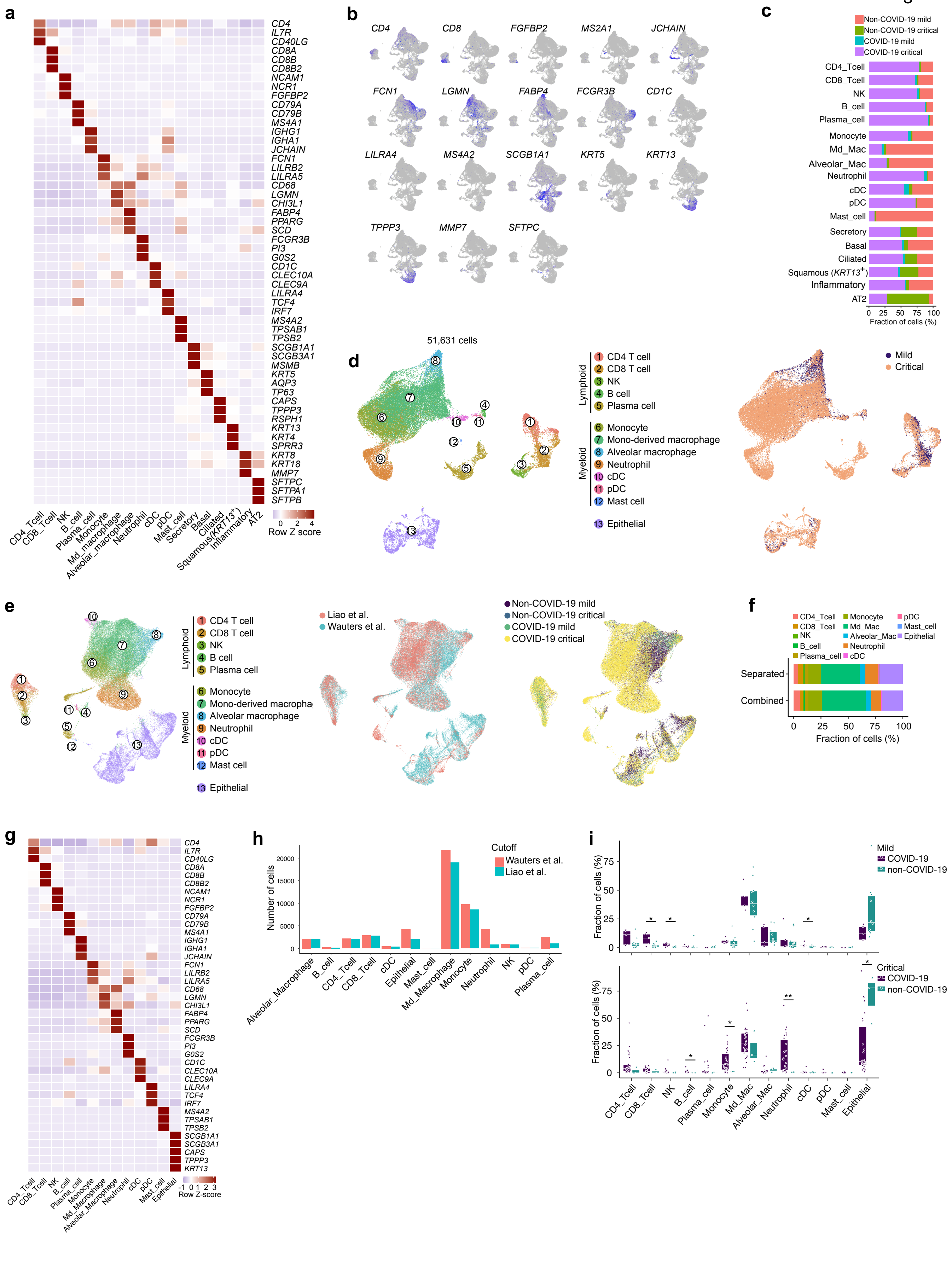

a
b
c
d
e
f
g
h
i
Row Z score
51,631 cells
Lymphoid
Myeloid
CD4 T cell
CD8 T cell
NK
B cell
Plasma cell
Monocyte
Mono-derived macrophage
Alveolar macrophage
Neutrophil
cDC
pDC
Mast cell
Epithelial
Mild
Critical
Non-COVID-19 mild
Non-COVID-19 critical
COVID-19 mild
COVID-19 critical
Fraction of cells (%)
Liao et al.
Wauters et al.
Separated
Combined
Row Z-score
Cutoff
Number of cells
COVID-19
non-COVID-19

Fig. S1 Cell type annotation and clustering of scRNA-seq data by Liao et al.

a-b Heatmap showing the expression of 3 marker genes (**a**) and feature plots showing 1 marker gene (**b**) for each of the different cell types. **c** Fraction of cell type per disease (COVID-19 vs non-COVID-19) and severity of the disease (mild vs critical). **d** UMAP showing 51,631 cells identified in scRNA-seq data by Liao et al., color-coded per cell type (left) and per disease severity status (right). **e** UMAP representation of merged datasets (Liao et al., and Wauters et al.) by scRNA-seq color-coded for the indicated cell type (left), the dataset (middle) and the disease (right). **f** Fraction of each cell type as determined by clustering both datasets separately or together. **g** Heatmap showing cell types with corresponding marker genes and functional gene sets. **h** Number of cells detected for each cell type using two quality cutoffs: the cutoff described in Wauters et al. vs the cutoff described in Liao et al. **i** Relative contribution of each cell type (in %) in COVID-19 vs non-COVID-19 in mild (upper panel) and critical disease (lower panel).
