# Supplementary material for: Discriminating mild from critical COVID-19 by innate and adaptive immune single-cell profiling of bronchoalveolar lavages
Source: Cell Res. 2021 Jan 21;31(3):272–90. doi: 10.1038/s41422-020-00455-9 (PMC8027624; doi:10.1038/s41422-020-00455-9)
Supplement: Supplementary file 2 — Supplementary Figure S2 [file 41422_2020_455_MOESM2_ESM.pdf]

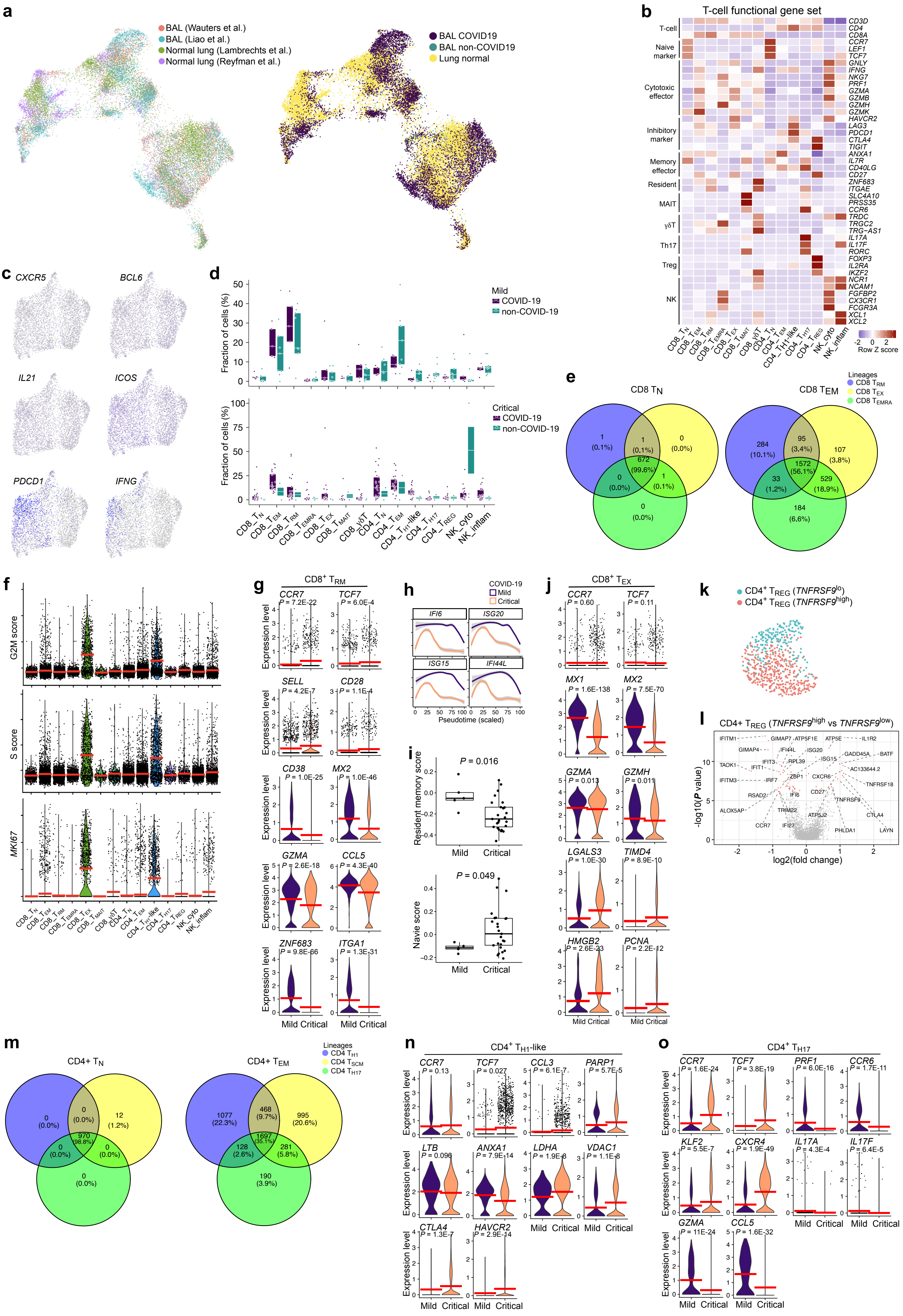
a
BAL (Wauters et al.)
BAL (Liao et al.)
Normal lung (Lambrechts et al.)
Normal lung (Reyfman et al.)
BAL COVID19
BAL non-COVID19
Lung normal
b
T-cell functional gene set
T-cell
Naive marker
Cytotoxic effector
Inhibitory marker
Memory effector
Resident
MAIT
γδT
Th17
Treg
NK
Row Z score
c
CXCR5
BCL6
IL21
ICOS
PDCD1
IFNG
d
Fraction of cells (%)
Mild
COVID-19
non-COVID-19
Critical
COVID-19
non-COVID-19
e
CD8 TN
CD8 TEM
Lineages
CD8 TRM
CD8 TEX
CD8 TEMRA
f
G2M score
S score
MKI67
g
CD8+ TRM
Expression level
h
COVID-19
Mild
Critical
IFI6
ISG20
ISG15
IFI44L
Pseudotime (scaled)
i
Resident memory score
P = 0.016
Mild
Critical
Navie score
P = 0.049
j
CD8+ TEX
k
CD4+ TREG (TNFRSF9lo)
CD4+ TREG (TNFRSF9high)
l
CD4+ TREG (TNFRSF9high vs TNFRSF9low)
-log10(P value)
log2(fold change)
m
CD4+ TN
CD4+ TEM
Lineages
CD4 TH1
CD4 TSCM
CD4 TH17
n
CD4+ TH1-like
o
CD4+ TH17

Fig. S2 Single-cell profiling of T-/NK-cell phenotypes

a UMAP showing 23,468 T-/NK-cells color-coded per data origin (left) and per COVID-19 disease status (right). **b** Heatmap showing T-/NK-cell phenotypes with corresponding marker genes and functional gene sets. **c** UMAP for the expression of key T_{FH} markers (*CXCR5*, *BCL6*, *IL21*, and *ICOS*) and other helper T-cell markers (*PDCD1*, *IFNG*), suggesting a lack of T_{FH}-cells. **d** Relative contribution of each cell type (in %) in mild (upper panel) and critical (lower panel) pneumonia patients comparing COVID-19 vs non-COVID-19. **e** Numbers and percentages of CD8+ T_N (left) and T_{EM} (right) T-cells shared by three $CD8^+$ T cell lineages. **f** Violin plot showing scores of cell cycle scores (G2M and S) and *MKI67* expression of T cell clusters. **g** Violin plot showing differential expression of genes in T-cells belonging to the $CD8^+$ T_{RM} lineage in mild vs critical COVID-19 patients. **h** Expression of interferon response genes along the $CD8^+$ T_{RM} trajectory. **i** Box plot showing 'resident memory' and 'naïve T-cell' scores of the CD8+ T_{RM} lineage from mild-moderate and critical patients. **j** Violin plot showing differential expression of genes in T-cells belonging to the $CD8^+$ T_{EX} lineage in mild vs critical COVID-19 patients. **k** UMAP of CD4+ T_{REG} subclusters. **l** Volcano plot showing differentially expressed genes between *TNFRSF9*^{high} and *TNFRSF9*^{low} T_{REG} subclusters. **m** Numbers and percentages of CD4+ T_{h1}-like (left) and T_{h17} (right) T-cells shared by three $CD4^+$ T cell lineages. **n** Violin plot showing differential expression of genes in T-cells belonging to the $CD4^+$ T_{H1}-like lineage in mild vs critical COVID-19 patients. **o** Violin plot showing differential expression of genes in T-cells belonging to the $CD4^+$ T_{H17} lineage in mild vs critical COVID-19 patients.
