# Supplementary material for: Discriminating mild from critical COVID-19 by innate and adaptive immune single-cell profiling of bronchoalveolar lavages
Source: Cell Res. 2021 Jan 21;31(3):272–90. doi: 10.1038/s41422-020-00455-9 (PMC8027624; doi:10.1038/s41422-020-00455-9)
Supplement: Supplementary file 3 — Supplementary Figure S3 [file 41422_2020_455_MOESM3_ESM.pdf]

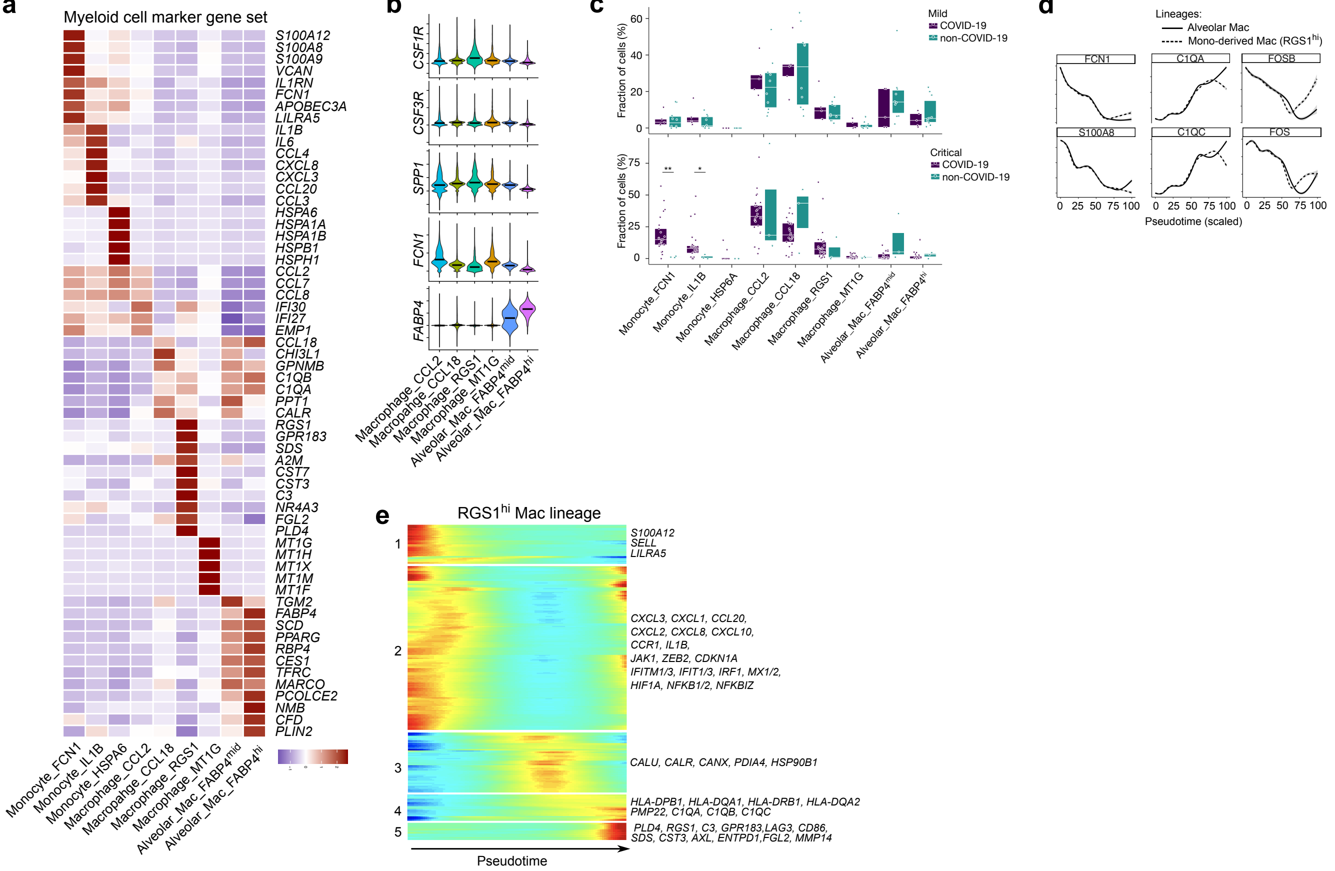

Fig. S3 Single-cell profiling of myeloid cells

a Heatmap showing myeloid cell phenotypes with corresponding marker gene sets. **b** Violin plots of normalized marker gene expression, distinguishing 6 macrophage subclusters based on their monocyte-derived characteristics, as indicated by monocyte lineage associated genes (*CSF1R*, *CSF3R*, *SPP1*, *FCN1*), and alveolar macrophage marker gene (*FABP4*). **c** Relative contribution of each cell type (in %) in mild (upper panel) and critical (lower panel) pneumonia patients comparing COVID-19 vs non-COVID-19. **d** Expression dynamics of key functional genes along the monocyte-to-macrophage trajectory. **e** Gene expression dynamics along the RGS1^{high} macrophage lineage. Genes cluster into 5 gene sets, each of them characterized by specific expression profiles, as depicted by a selection of marker gene characteristic for each set.
