# Supplementary material for: Discriminating mild from critical COVID-19 by innate and adaptive immune single-cell profiling of bronchoalveolar lavages
Source: Cell Res. 2021 Jan 21;31(3):272–90. doi: 10.1038/s41422-020-00455-9 (PMC8027624; doi:10.1038/s41422-020-00455-9)
Supplement: Supplementary file 4 — Supplementary Figure S4 [file 41422_2020_455_MOESM4_ESM.pdf]

Fig. S4

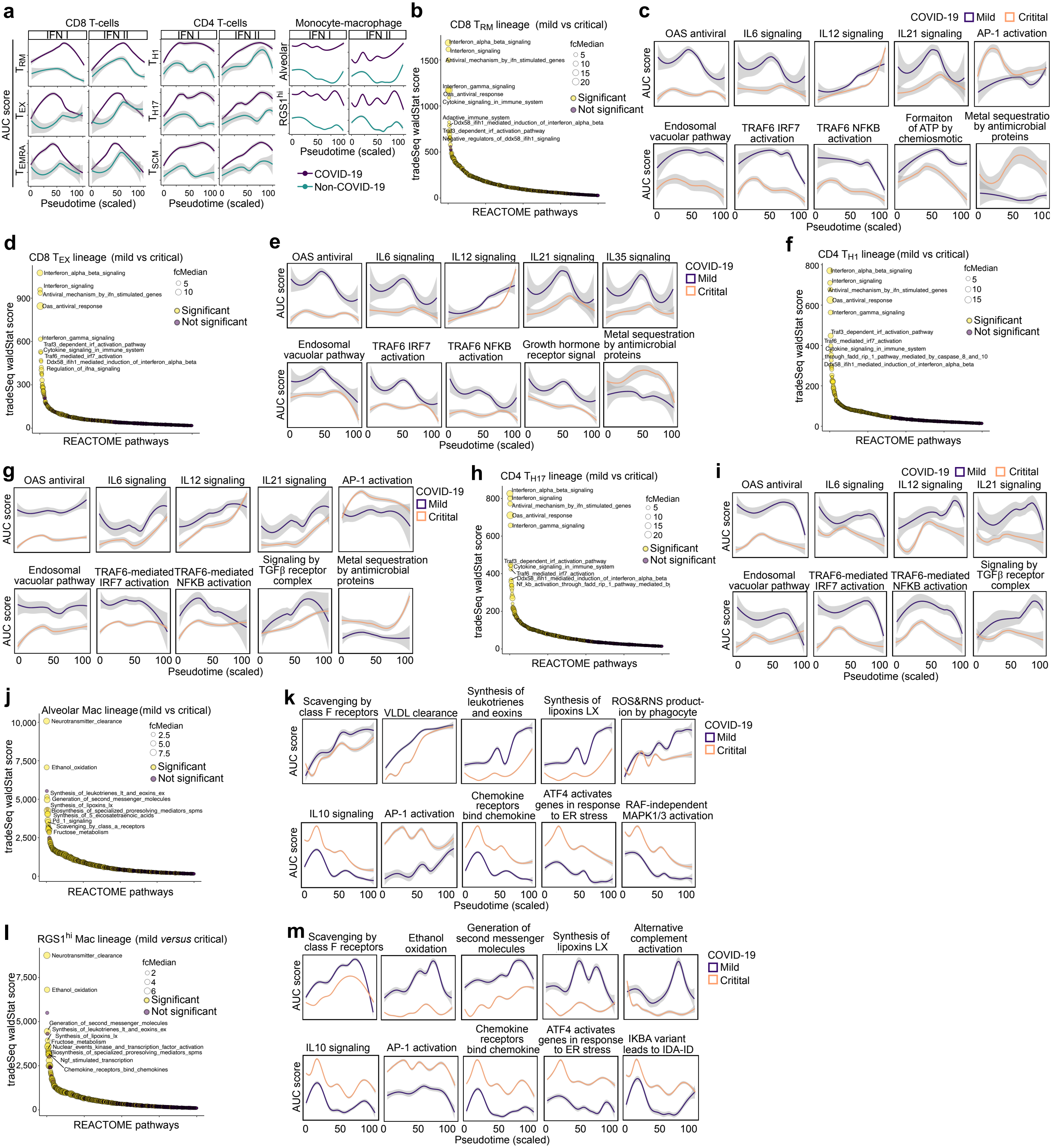
a
CD8 T-cells
CD4 T-cells
Monocyte-macrophage
IFN I
IFN II
AUC score
T_RM
T_EX
T_EMRA
T_H1
T_H17
T_SCM
Alveolar
RGS1hi
Pseudotime (scaled)
COVID-19
Non-COVID-19
b
CD8 T_RM lineage (mild vs critical)
tradeSeq waldStat score
REACTOME pathways
fcMedian
Significant
Not significant
Interferon_alpha_beta_signaling
Interferon_signaling
Antiviral_mechanism_by_ifn_stimulated_genes
Interferon_gamma_signaling
Oas_antiviral_response
Cytokine_signaling_in_immune_system
Adaptive_immune_system
Ddx58_ifih1_mediated_induction_of_interferon_alpha_beta
Traf3_dependent_irf_activation_pathway
Negative_regulators_of_ddx58_ifih1_signaling
c
COVID-19
Mild
Critital
OAS antiviral
IL6 signaling
IL12 signaling
IL21 signaling
AP-1 activation
Endosomal vacuolar pathway
TRAF6 IRF7 activation
TRAF6 NFKB activation
Formaiton of ATP by chemiosmotic
Metal sequestratio by antimicrobial proteins
d
CD8 T_EX lineage (mild vs critical)
Regulation_of_ifna_signaling
Traf6_mediated_irf7_activation
e
IL35 signaling
Growth hormone receptor signal
Metal sequestration by antimicrobial proteins
f
CD4 T_H1 lineage (mild vs critical)
Cytokine_signaling_in_immune_system
through_fadd_rip_1_pathway_mediated_by_caspase_8_and_10
g
TRAF6-mediated IRF7 activation
TRAF6-mediated NFKB activation
Signaling by TGFβ receptor complex
h
CD4 T_H17 lineage (mild vs critical)
Nf_kb_activation_through_fadd_rip_1_pathway_mediated_by
i
j
Alveolar Mac lineage (mild vs critical)
Neurotransmitter_clearance
Ethanol_oxidation
Synthesis_of_leukotrienes_lt_and_eoxins_ex
Generation_of_second_messenger_molecules
Synthesis_of_lipoxins_lx
Biosynthesis_of_specialized_proresolving_mediators_spms
Synthesis_of_5_eicosatetraenoic_acids
Pd_1_signaling
Scavenging_by_class_a_receptors
Fructose_metabolism
k
Scavenging by class F receptors
VLDL clearance
Synthesis of leukotrienes and eoxins
Synthesis of lipoxins LX
ROS&RNS production by phagocyte
IL10 signaling
Chemokine receptors bind chemokine
ATF4 activates genes in response to ER stress
RAF-independent MAPK1/3 activation
l
RGS1hi Mac lineage (mild versus critical)
Nuclear_events_kinase_and_transcription_factor_activation
Ngf_stimulated_transcription
Chemokine_receptors_bind_chemokines
m
Ethanol oxidation
Generation of second messenger molecules
Alternative complement activation
IKBA variant leads to IDA-ID

Fig. S4 Pathway analysis along T-cell and monocyte-to-macrophage lineages

a Differentially-activated IFN I and II pathways along the $CD8^+$, $CD4^+$ and monocyte-to-macrophage lineages, comparing COVID-19 vs non-COVID-19. **b** Differentially-activated REACTOME pathways for the $CD8^+$ T_{RM}-lineage, comparing mild vs critical COVID-19. **c** A selection of 10 differentially-activated REACTOME pathways along the $CD8^+$ T_{RM}-lineage, comparing mild vs critical COVID-19. **d** Differentially-activated REACTOME pathways along the $CD8^+$ T_{EX}-lineage, comparing mild vs critical COVID-19. **e** A selection of 10 differentially-activated REACTOME pathways along the $CD8^+$ T_{ex}-lineage, comparing mild vs critical COVID-19. **f** Differentially-activated REACTOME pathways for the $CD4^+$ T_{H1}-lineage, comparing mild vs critical COVID-19. **g** A selection of 10 differentially-activated REACTOME pathways along the $CD4^+$ T_{H1}-lineage, comparing mild vs critical COVID-19. **h** Differentially-activated REACTOME pathways for the $CD4^+$ T_{H17}-lineage, comparing mild vs critical COVID-19. **i** A selection of 8 differentially-activated REACTOME pathways along the $CD4^+$ T_{H17}-lineage, comparing mild vs critical COVID-19. **j** Differentially-activated REACTOME pathways for the alveolar macrophage lineage, comparing mild vs critical COVID-19. **k** A selection of 10 differentially-activated REACTOME pathways along the alveolar macrophage lineage, comparing mild vs critical COVID-19. **l** Differentially-activated REACTOME pathways for the RGS1-macrophage lineage, comparing mild vs critical COVID-19. **m** A selection of 10 differentially-activated REACTOME pathways along the RGS1-macrophage lineage, comparing mild vs critical COVID-19.
