# Supplementary material for: Discriminating mild from critical COVID-19 by innate and adaptive immune single-cell profiling of bronchoalveolar lavages
Source: Cell Res. 2021 Jan 21;31(3):272–90. doi: 10.1038/s41422-020-00455-9 (PMC8027624; doi:10.1038/s41422-020-00455-9)
Supplement: Supplementary file 5 — Supplementary Figure S5 [file 41422_2020_455_MOESM5_ESM.pdf]

Fig. S5

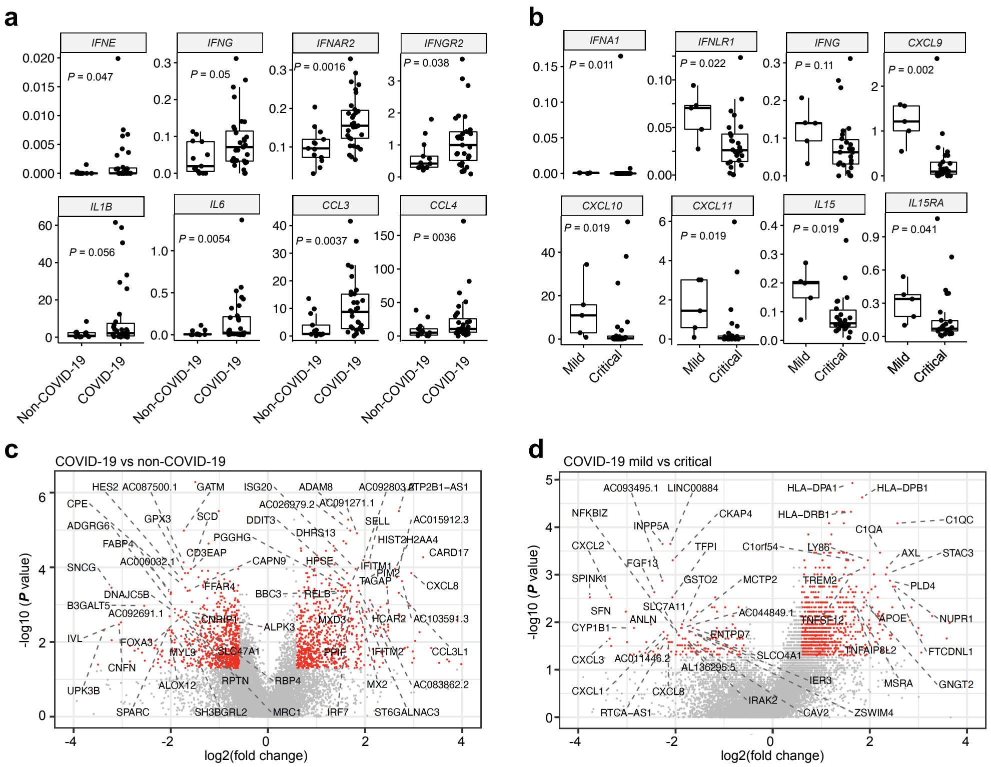

Figure S5 Differentially expressed genes in different diseases and conditions
a Among all 27 interferon genes and their receptors detected in the scRNA-seq dataset, as well as several key chemokines/cytokines (*CXCL9*, *CXCL10*, *CXCL11*, *CCL3*, *CCL4*, *CCL8*, *IL6*, *IL1B*, *IL15*, *IL15RA*) related to anti-viral responses. Box plots showing differentially expressed genes in non-COVID-19 vs COVID-19 patients (calculated per patient, $P <= 0.05$ expect for *IL1B*). **b** Box plots showing differentially expressed interferon and receptor genes, as well as related cytokines in mild vs critical COVID-19 patients (calculated per patient, $P < 0.05$ expect for *IFNG*). **c** Volcano plot of differentially expressed genes comparing COVID-19 vs non-COVID-19 patients (calculated per patient). Red dots, $P < 0.05$. **d** Volcano plot of differentially expressed genes comparing COVID-19 mild vs critical patients (calculated per patient). Red dots, $P < 0.05$.
