# Supplementary material for: Discriminating mild from critical COVID-19 by innate and adaptive immune single-cell profiling of bronchoalveolar lavages
Source: Cell Res. 2021 Jan 21;31(3):272–90. doi: 10.1038/s41422-020-00455-9 (PMC8027624; doi:10.1038/s41422-020-00455-9)
Supplement: Supplementary file 6 — Supplementary Figure S6 [file 41422_2020_455_MOESM6_ESM.pdf]

Fig. S6

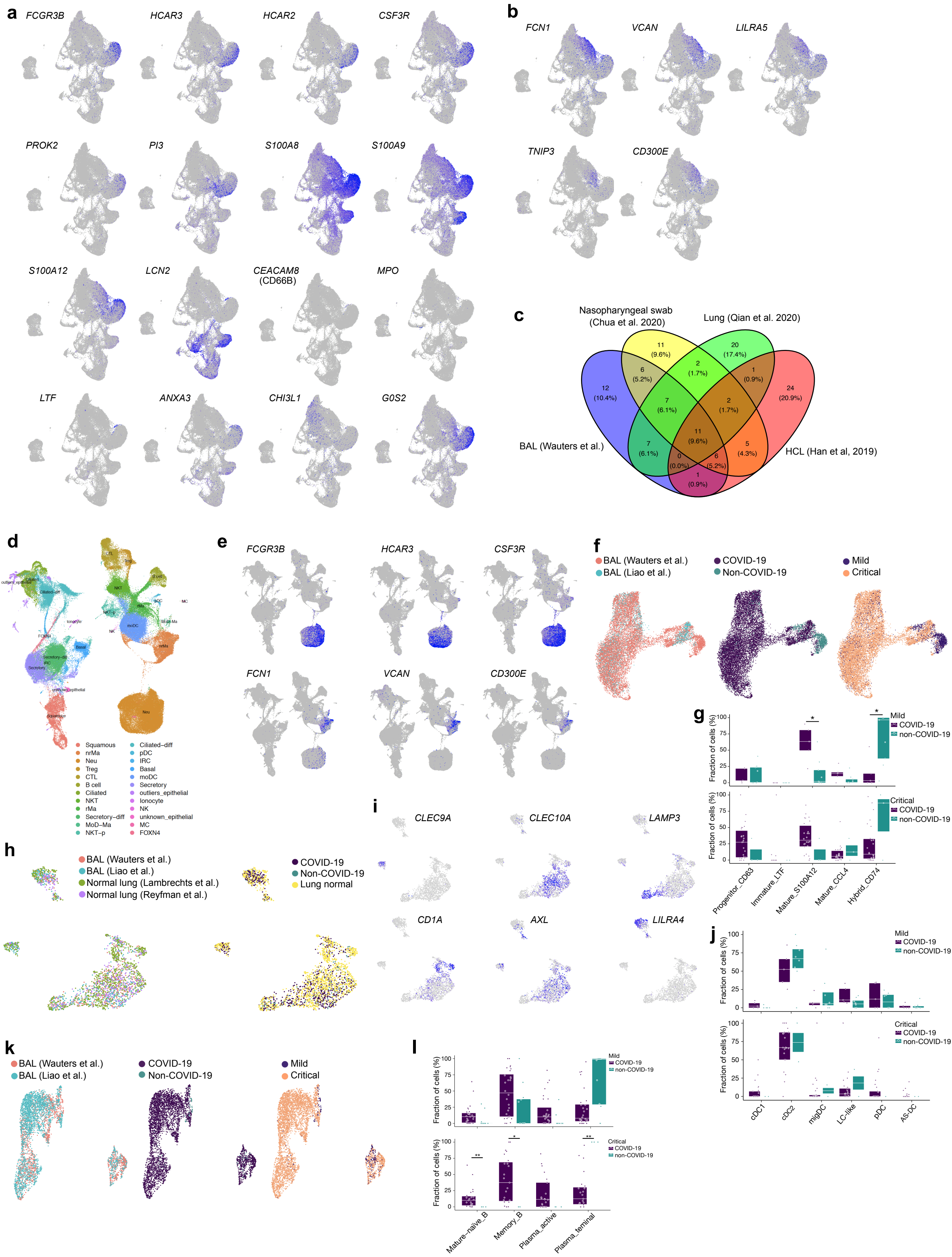
a
FCGR3B
HCAR3
HCAR2
CSF3R
PROK2
PI3
S100A8
S100A9
S100A12
LCN2
CEACAM8 (CD66B)
MPO
LTF
ANXA3
CHI3L1
G0S2
b
FCN1
VCAN
LILRA5
TNIP3
CD300E
c
Nasopharyngeal swab (Chua et al. 2020)
Lung (Qian et al. 2020)
BAL (Wauters et al.)
HCL (Han et al, 2019)
11 (9.6%)
20 (17.4%)
6 (5.2%)
2 (1.7%)
1 (0.9%)
12 (10.4%)
7 (6.1%)
2 (1.7%)
24 (20.9%)
11 (9.6%)
7 (6.1%)
0 (0.0%)
6 (5.2%)
5 (4.3%)
1 (0.9%)
d
Squamous
nrMa
Neu
Treg
CTL
B cell
Ciliated
NKT
rMa
Secretory–diff
MoD–Ma
NKT–p
Ciliated–diff
pDC
IRC
Basal
moDC
Secretory
outliers_epithelial
Ionocyte
NK
unknown_epithelial
MC
FOXN4
e
FCGR3B
HCAR3
CSF3R
FCN1
VCAN
CD300E
f
BAL (Wauters et al.)
BAL (Liao et al.)
COVID-19
Non-COVID-19
Mild
Critical
g
Fraction of cells (%)
Mild
COVID-19
non-COVID-19
Critical
COVID-19
non-COVID-19
Progenitor_CD63
Immature_LTF
Mature_S100A12
Mature_CCL4
Hybrid_CD74
h
BAL (Wauters et al.)
BAL (Liao et al.)
Normal lung (Lambrechts et al.)
Normal lung (Reyfman et al.)
COVID-19
Non-COVID-19
Lung normal
i
CLEC9A
CLEC10A
LAMP3
CD1A
AXL
LILRA4
j
Fraction of cells (%)
Mild
COVID-19
non-COVID-19
Critical
COVID-19
non-COVID-19
cDC1
cDC2
migDC
LC-like
pDC
AS-DC
k
BAL (Wauters et al.)
BAL (Liao et al.)
COVID-19
Non-COVID-19
Mild
Critical
l
Fraction of cells (%)
Mild
COVID-19
non-COVID-19
Critical
COVID-19
non-COVID-19
Mature–naive_B
Memory_B
Plasma_active
Plasma_terminal

Fig. S6 Neutrophil, dendritic, B- and epithelial cell phenotyping in COVID-19

a Feature plots showing expression of neutrophil marker genes and related genes. **b** Feature plots showing expression of monocyte marker genes. **c** In the 4 indicated studies we selected the 50 most specific neutrophil markers and we calculated the overlap of the resulting markers (n=115) between each study. **d** UMPA of the Chua et al. data showing all cell types detected in COVID-19 nasopharyngeal and bronchial samples. **e** The neutrophil (upper panel) and monocyte (lower panel) marker genes expressed in the cell types of (d). **f** UMAP showing 14,154 neutrophils color-coded per data origin (left), per disease status (middle) and per disease severity status (right). **g** Relative contribution of each neutrophil cell type (in %) in mild (upper panel) and critical (lower panel) pneumonia patients comparing COVID-19 vs non-COVID-19. **h** UMAP of 1,410 DCs, color-coded per data origin (left) and per disease status (right). **i** Feature plots of marker genes, distinguishing 6 DC subclusters: type I classical dendritic cells (*CLEC9A*), type II classical dendritic cells (*CLEC10A*), migratory dendritic cells (*LAMP3*), Langerhans cell-like dendritic cells (*CD1A*), AS-DC (*AXL*) and plasmacytoid dendritic cells (*LILRA4*). **j** Relative contribution of each DC cell type (in %) in mild (upper panel) and critical (lower panel) pneumonia patients comparing COVID-19 vs non-COVID-19. **k** UMAP of 1,397 B-cells, color-coded per data origin (left), per disease status (middle) and per disease severity status (right). **l** Relative contribution of each B and plasma cell type (in %) in mild (upper panel) and critical (lower panel) pneumonia patients comparing COVID-19 vs non-COVID-19.
