# Supplementary material for: Discriminating mild from critical COVID-19 by innate and adaptive immune single-cell profiling of bronchoalveolar lavages
Source: Cell Res. 2021 Jan 21;31(3):272–90. doi: 10.1038/s41422-020-00455-9 (PMC8027624; doi:10.1038/s41422-020-00455-9)
Supplement: Supplementary file 7 — Supplementary Figure S7 [file 41422_2020_455_MOESM7_ESM.pdf]

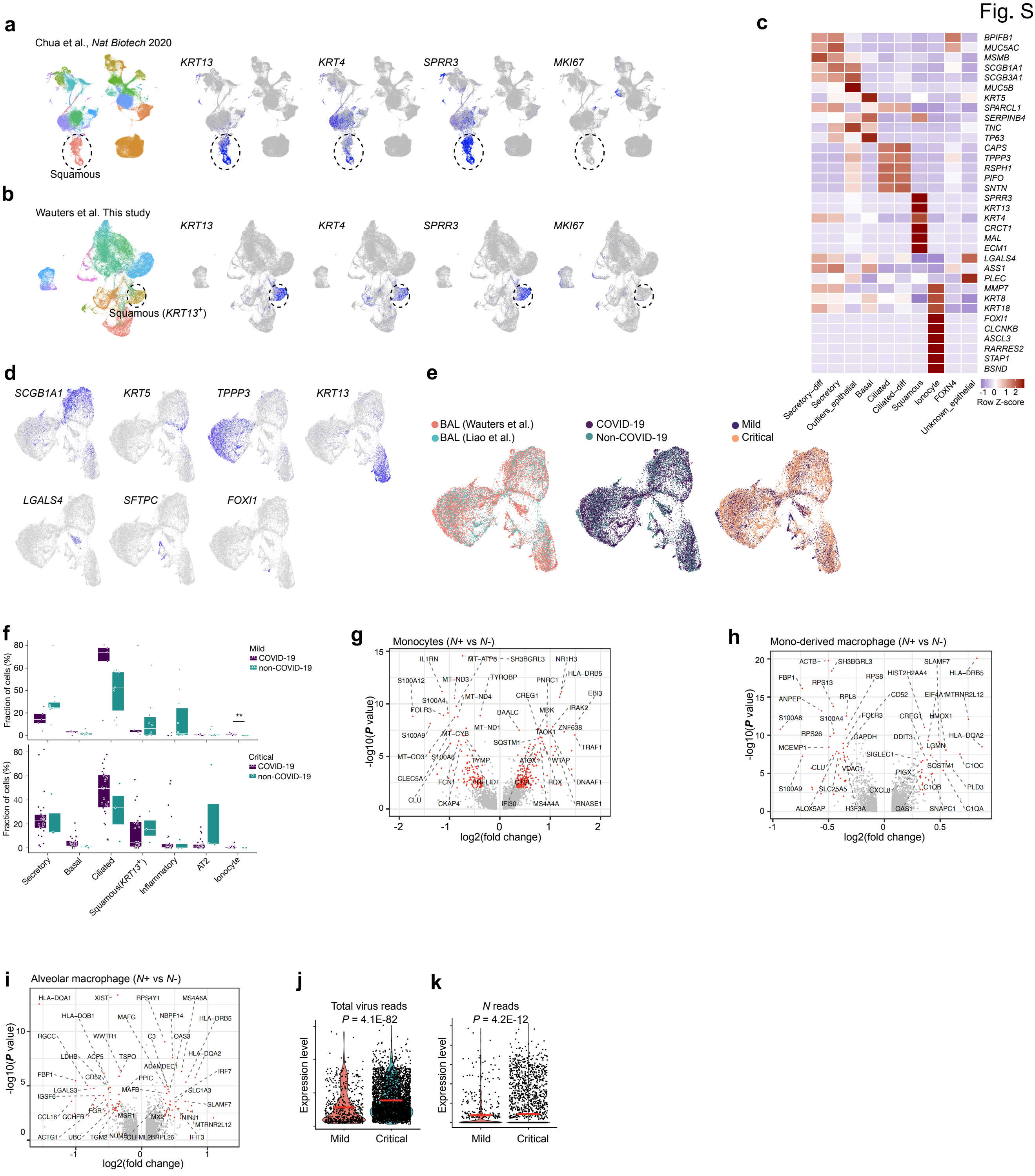

Figure S7. Epithelial cell types and viral-host interaction

a UMAP from Chua et al. showing marker gene expression (*KTR13*, *KRT4* and *SPRR3*) and *MKI67* expression in a subset of epithelial cells. **b** Expression of these genes in the epithelial cells identified by Wauters et al. **c** Heatmap of epithelial marker genes defined in Fig.7b (AT2 markers excluded) plotted for epithelial cell types defined in Chua et al. dataset. **d** Feature plots of marker genes, distinguishing 7 epithelial cell subclusters: secretory cells (*SCGB1A1*), basal cells (*KRT5*), ciliated cells (*TPPP3*), squamous cells (*KRT13*), inflammatory cells (*LGALS4*), alveolar type II cells (*SFTPC*) and ionocytes (*FOXI1*). **e** UMAP of 22,215 epithelial cells, color-coded per data origin (left), per disease status (middle) and per disease severity status (right). **f** Relative contribution of each epithelial cell type (in %) in mild (upper panel) and critical (lower panel) pneumonia patients comparing COVID-19 vs non-COVID-19. **g-i** Differentially expressed genes in *N*+ vs *N*- monocytes (**g**), monocyte-derived macrophages (**h**) and alveolar macrophages (**i**). **j-k** Violin plots comparing the number of reads mapping to the complete viral genome (**j**) and the *N* sequence of the viral genome (**k**) between mild and critical COVID-19 patients.
