# Supplementary material for: Discriminating mild from critical COVID-19 by innate and adaptive immune single-cell profiling of bronchoalveolar lavages
Source: Cell Res. 2021 Jan 21;31(3):272–90. doi: 10.1038/s41422-020-00455-9 (PMC8027624; doi:10.1038/s41422-020-00455-9)
Supplement: Supplementary file 8 — Supplementary Figure S8 [file 41422_2020_455_MOESM8_ESM.pdf]

Fig. S8

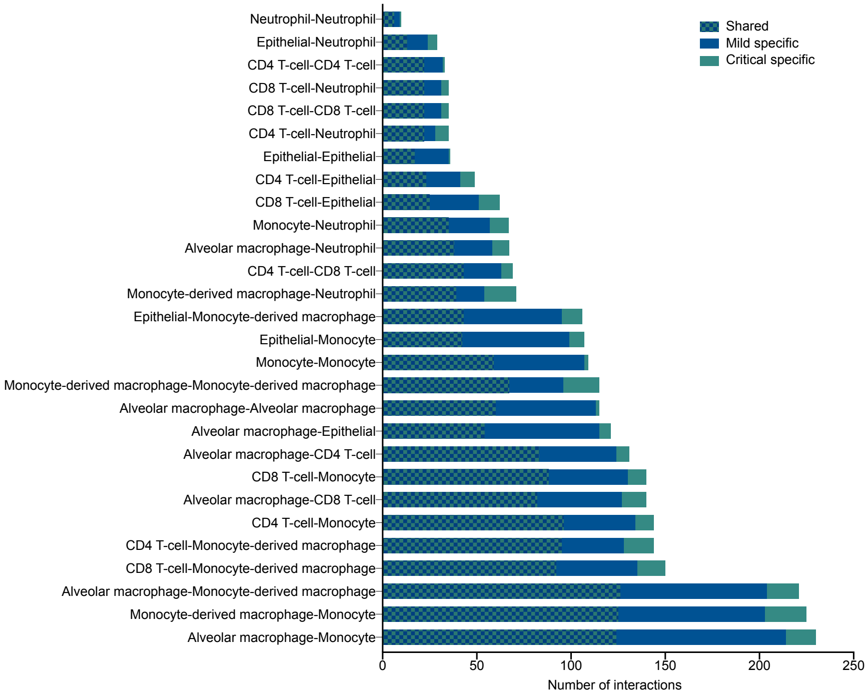

Figure S8. Cell-to-cell interactions shared and specific in mild and critical COVID-19 BAL
Predicted number of cell-to-cell interactions between epithelial cell, neutrophil, monocyte, macrophage, $CD8^+/CD4^+$ T-cell from BAL of mild vs critical COVID-19.
