# Supplementary material for: Discriminating mild from critical COVID-19 by innate and adaptive immune single-cell profiling of bronchoalveolar lavages
Source: Cell Res. 2021 Jan 21;31(3):272–90. doi: 10.1038/s41422-020-00455-9 (PMC8027624; doi:10.1038/s41422-020-00455-9)
Supplement: Supplementary file 9 — Supplementary Figure S9 [file 41422_2020_455_MOESM9_ESM.pdf]

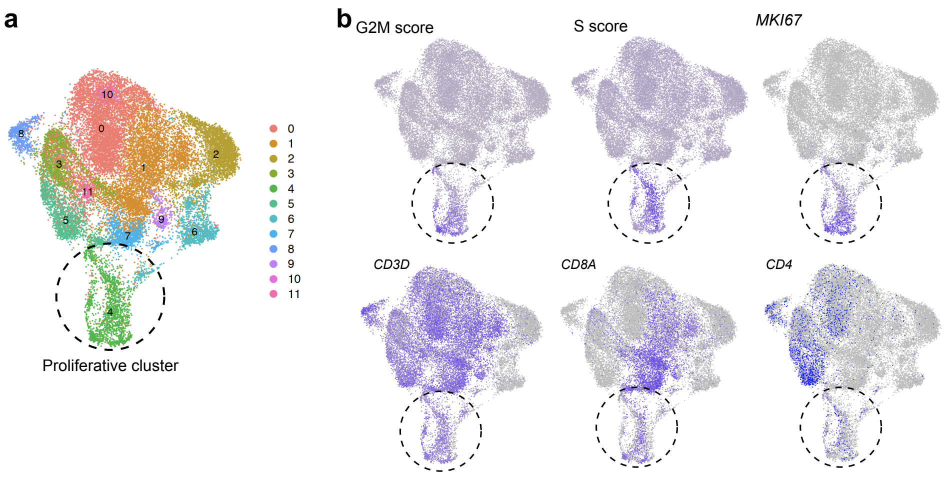

Figure S9. T cell clustering without regressing out cell cycle effect

a UMAP of T cell clusters without regressing our cell cycle. Cluster 4 is a proliferative cluster. **b** UMAP of T cells showing cell cycle scores (G2M and S) and key marker genes.
