# Supplementary material for: Discriminating mild from critical COVID-19 by innate and adaptive immune single-cell profiling of bronchoalveolar lavages
Source: Cell Res. 2021 Jan 21;31(3):272–90. doi: 10.1038/s41422-020-00455-9 (PMC8027624; doi:10.1038/s41422-020-00455-9)
Supplement: Supplementary file 10 — Supplementary Table S1 [file 41422_2020_455_MOESM10_ESM.pdf]

Supplementary Table S1. Demographics and characteristics of study cohort.

	Mild COVID-19 (n=2)	Critical COVID-19 (n=20)	Mild non-COVID pneumonia (n=10)	Critical non-COVID pneumonia (n=3)
Age, years[a]	63 [55-71]	60 [54.5-68]	67.5 [58.75-72.5]	73 [72-74.5]
Sex	..	..	..	..
Men	1 (50)	15 (75)	5 (50)	2 (67)
Women	1 (50)	5 (25)	5 (50)	1 (33)
Co-morbid conditions				
Diabetes mellitus type II	1 (50)	3 (15)	1 (10)	1 (33)
Arterial hypertension	2 (100)	14 (70)	5 (50)	3 (100)
Medication history	..	..	..	..
Immunosuppressants	1 (50)[b]	0 (0)	4 (40)[c]	0 (0)
CT severity score[d]	7.5 [7-8]	16 [12-23][e]	3.5 [1-7.5]	6 [6-11.5]
Time from illness onset to sampling (days)	17.5 [15-20]	19 [15.75-25]	10.5 [2.5-14.75]	9 [5-19]
Bronchoalveolar lavage microbiology	..	..	..	..
SARS-CoV-2 PCR positive	2 (100)	7 (35)[f]	0 (0)	0 (0)
Other viral PCR positive	0 (0)	4 (20)[g]	2 (20)[h]	1 (33)[i]
Bacterial culture positive	1 (50)[j]	3 (15)[k]	1 (10)[l]	1 (33)[m]
PJP PCR positive	0 (0)	0 (0)	5 (50)	0 (0)
Respiratory support	2 (100)	20 (100)	8 (80)	3 (100)
Oxygen via nasal cannula	2 (100)	0 (0)	8 (80)	0 (0)
High-flow oxygen therapy	0 (0)	0 (0)	0 (0)	1 (33)
Mechanical ventilation	0 (0)	15 (75)	0 (0)	2 (67)
Extracorporeal membrane oxygenation	0 (0)	5 (25)	0 (0)	0 (0)
Medical treatment	2 (100)	20 (100)	7 (70)	3 (100)
Antiviral therapy (<7d)	0 (0)	14 (70)[n]	0 (0)	0 (0)
Antibiotics (<7d)	2 (100)	20 (100)	7 (70)	3 (100)
Immunomodulatory therapy (<7d)	0 (0)	5 (25)[o]	0 (0)	0 (0)
Fatal outcome[p]	0 (0)	1 (5)	1 (10)	1 (33)

Legend to Supplementary Table S1: Data are median [IQR], median [range] for the "Mild COVID-19" group or n (%). [a] Prespecified subgroups were well matched demographically, except for a significant older age of critical non-COVID compared to COVID-19 patients. Statistical analyses are shown in Table S11. [b] 1 patient received chemotherapy. [c] 2 patients received chemotherapy, 1 patient taking abemaciclib and 1 patient taking mycophenolate mofetil/tacrolimus. [d] A CT score was assigned by converting percentage of lung parenchyma opacity for each lobe into a 5-points Likert scale: a score of 0 for 0% lung opacity (LO), 1 for 1% to <5% LO, 2 for 5- 25% LO, 3 for 26-50% LO, 4 for 51-75% LO and 5 for 76-100 LO. The total CT score is the sum of the individual lobar scores. COVID-19 disease status and disease severity both independently predicted CT Score, as assessed by logistic regression (P=0,047 and P=0,024 respectively (Wald test)). [e] No chest CT available for 3 patients. [f] SARS-CoV-2 PCR not performed in 12 cases and 1 negative SARS-CoV-2 PCR on BAL (after initial

PCR-confirmed diagnosis of COVID-19). [g] Herpes Simplex 1 PCR positive in 4 cases. [h] Herpes Simplex 1 PCR positive in 2 cases, 1 case of PCR-confirmed coronavirus HKU-1 co-infection. [i] Herpes Simplex 1 PCR positive in 1 case. [j] 1 patient with positive *Escherichia coli* and *Pseudomonas aeruginosa* culture. [k] 3 patients with cultures positive for *Raoultella ornithinolytoca, Klebsiella aerogenes* or *Enterobacter cloacae*. [l] 1 patient with cultures positive for *Escherichia coli;* in 4 patients, organism remained unidentified, 2 of them received prior antibiotics. [m] 1 patient with cultures positive for *Pseudomonas aeruginosa*; in 2 patients, organism remained unidentified, 2 of them received prior antibiotics. [n] Hydroxychloroquine administered in 12 cases, remdesivir administered in 2 cases. [o] 5 patients received >1mg/kg prednisone during 48h preceding sampling; 1 patient received Anakinra during the week preceding sampling. [p] Recruitment started on March 27th 2020, with outcome assessed on June 11th 2020. PJP: Pneumocystis jirovecii; SARS-CoV-2: Severe acute respiratory syndrome coronavirus 2.
